# When collagen fails: Zinc isotopes unlock Sumerian lifeways in southern Mesopotamia

**DOI:** 10.1073/pnas.2526276123

**Published:** 2026-03-09

**Authors:** Matteo Giaccari, Licia Romano, Silvia Soncin, Sofia Panella, Francesca Alhaique, Franco D’Agostino, Klervia Jaouen, Mary Anne Tafuri

**Affiliations:** ^a^Department of Earth Sciences, Sapienza Università di Roma, Rome 00185, Italy; ^b^School of Historical and Philosophical Studies, Faculty of Arts, University of Melbourne, Parkville, Victoria 3010, Australia; ^c^Department Italian Institute of Oriental Studies, Sapienza University of Rome, Rome 00185, Italy; ^d^Department of Environmental Biology, Sapienza Università di Roma, Rome 00185, Italy; ^e^Bioarchaeology Service, Museo delle Civiltà, Rome 00144, Italy; ^f^Géosciences Environnement Toulouse, Centre National de la Recherche Scientifique UMR 5563, Observatoire Midi-Pyrénées, Toulouse 31400, France

**Keywords:** ancient diet, human–animal interactions, multiproxy isotope analysis, carbon and oxygen isotopes, weaning practices

## Abstract

Reconstructing past lifeways and diets is essential to understanding the emergence of urban societies. However, in what are now arid environments like southern Mesopotamia, poor collagen preservation has long hampered direct isotopic analysis of trophic levels. This limitation has left key gaps in our understanding of subsistence in one of the world’s earliest urban heartlands. Here, we apply zinc isotope analysis to human and faunal dental enamel from the third-millennium BCE site of Abu Tbeirah (Iraq), integrating δ^13^C, δ^18^O, and trace element ratios (Ba/Ca and Sr/Ca). This multiproxy approach reveals an omnivorous diet based on C_3_ cereals, terrestrial animal products (likely including pigs), and limited freshwater resources, with no or little evidence of marine fish consumption, despite the site’s proximity to the ancient shoreline. Dietary patterns do not vary by sex, suggesting broad access to similar food sources within this nonelite population. Moreover, zinc and carbon isotopes proved valuable in identifying animal feeding practices. Our results provide direct dietary evidence from southern Mesopotamia, overcoming long-standing preservation challenges. The results allow us to evaluate specific expectations about diet and animal management in a collagen-poor context, also highlighting early-life feeding behaviors. They demonstrate the power of zinc isotopes to reconstruct trophic level in collagen-poor contexts, opening broad avenues for bioarchaeological research in early complex societies.

## Significance

Understanding ancient diets is one of the keys to reconstructing lifeways and social structures. In what are now arid regions like southern Mesopotamia, poor collagen preservation has long hindered direct dietary reconstructions. Here, we apply zinc isotope analysis to human and faunal dental enamel from the third-millennium BCE site of Abu Tbeirah (southern Iraq), offering a method to overcome this limitation. Combined with carbon and oxygen isotopes and trace element ratios (Ba/Ca and Sr/Ca), zinc isotopes reveal an omnivorous diet based on C_3_ cereals, terrestrial animal protein, and possibly freshwater resources, with no evidence of marine fish consumption. These findings offer individual-level insight into subsistence practices, early-life nutrition, and animal management within a nonelite population in early-urbanized southern Mesopotamia.

Isotopic proxies allow us to infer trophic level, plant use, and even weaning behavior directly from human tissues, not only at the population scale but also at the individual level (1–5). Beyond identifying subsistence strategies, isotope techniques reshaped our understanding of social and gender inequalities (6, 7), cultural changes (8), and land use (9, 10) in prehistoric and early historical contexts.

Yet these techniques face persistent limitations. Taphonomic and diagenetic processes, as well as environmental and anthropogenic factors, can alter the isotopic signatures preserved in bone and enamel, or hinder the biogenic composition of such tissues altogether. These limitations, while often underestimated, remain critical in determining where and how isotopic methods can be applied meaningfully.

In southern Mesopotamia, one of the world’s earliest urbanized regions, organic preservation is severely compromised by arid conditions, high soil salinity, and widespread bitumen contamination. As a result, traditional collagen-based isotope analysis is virtually impossible (11, 12). At Abu Tbeirah, only 3 out of 48 samples analyzed in previous studies yielded reliable collagen [(12) and *SI Appendix*, Text 2.5]. Even in northern Mesopotamia, which has yielded stable isotope data from archaeological human remains (13–16), environmental and agricultural factors, such as manuring and marsh ecosystems (even if smaller than those in the south), complicate interpretation (16).

Ancient Mesopotamia is known for its extensive corpus of administrative texts, which offer indirect glimpses into life in the region. Textual sources, archaeobotanical, and archeozoological evidence suggest diets centered on C_3_ cereals (barley and wheat) and secondary products such as bread, beer, dairy, and occasional meat or fish (13, 17–22). While pigs appear underrepresented in texts, some records suggest they were raised in urban contexts and fed on domestic by-products (23, 24). Access to meat and secondary products likely differentiated elite from nonelite diets (20). While such records are invaluable, they remain exceptional and, even in the case of texts on rations, do not provide complete evidence of the foods actually consumed by people (*SI Appendix*, Text 1). As a result, reconstructing diet at the individual level, rather than through generalized historical models, has been constrained in regions characterized by poor organic preservation and remains a major gap in southern Mesopotamian archaeology.

**Fig. 1.**
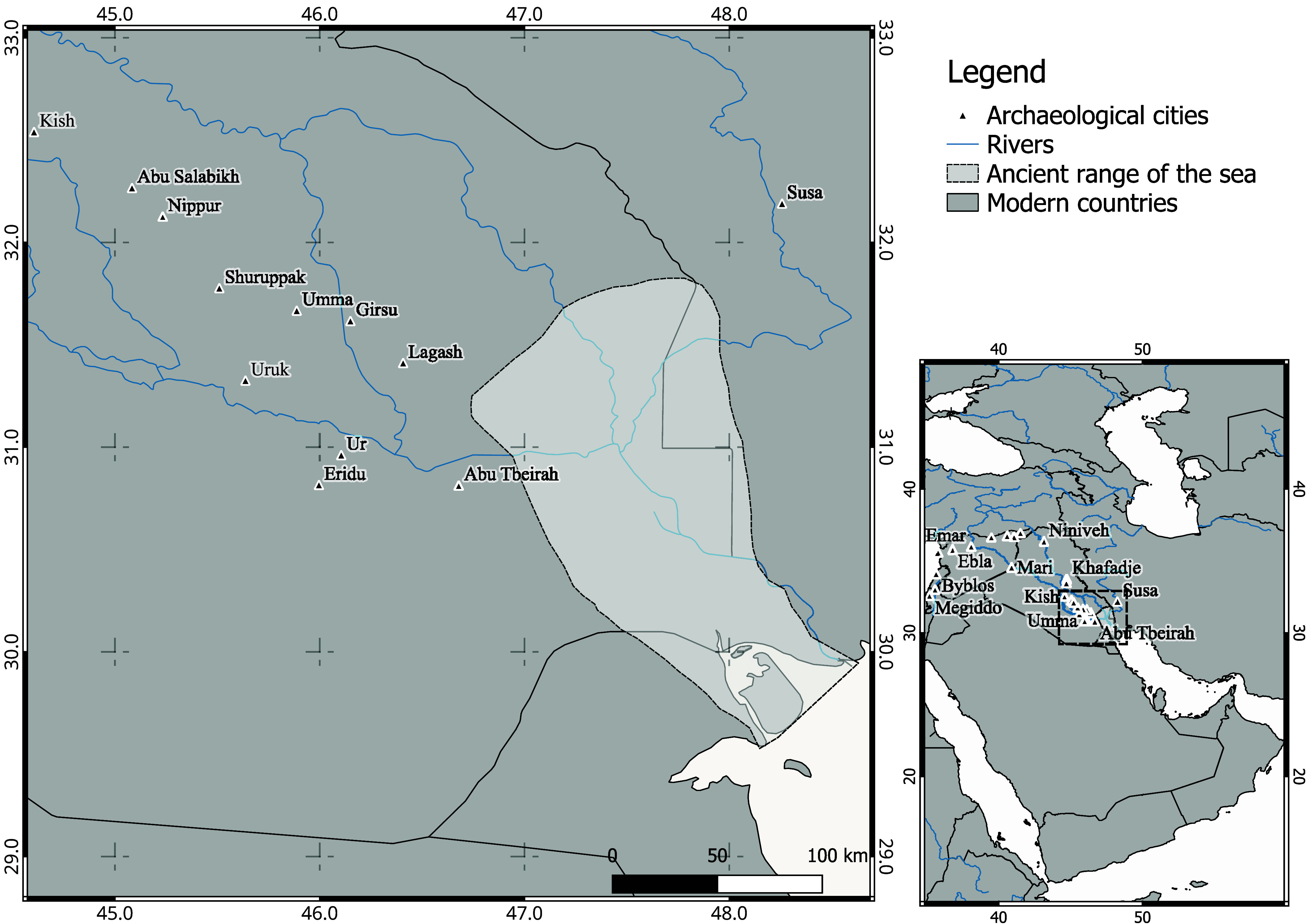
Topographic map of the Arabian Peninsula with a magnified view of ancient Mesopotamia, focusing on the southern and central regions.

In this sense, the third-millennium BCE site of Abu Tbeirah (Fig. 1), a medium-sized city near ancient Ur, gains importance. This city was mainly occupied during the Early Dynastic period (ED) and at the time was about 30 km from the Persian Gulf [*SI Appendix*, Text 2; and (17)]. The Abu Tbeirah burials are particularly valuable in the southern Mesopotamian contexts because they contain almost no prestigious grave goods or monumental structures (*SI Appendix*, Text 2.2). Hence, Abu Tbeirah provides the opportunity to document the daily lives of less privileged populations about whom historical sources generally say very little, regardless of period, as written evidence is primarily concerned with goods, practices, and activities relevant to elite administration and institutional management rather than everyday domestic experience. Our study seeks to explore the daily lives of nonelite populations and complement or even dispute historical documents, using an innovative isotopic approach that is compatible with arid environments.

Zinc isotopes (δ^66^Zn), preserved in dental enamel, offer a promising alternative to collagen-based isotopic analyses. Unlike nitrogen isotopes (δ^15^N), which require collagen, zinc can be measured in the inorganic matrix, namely in hydroxyapatite, which remains robust even in hostile burial environments (see e.g., refs. 25–27). A recent controlled feeding experiment study confirms that zinc isotope values of body tissues proportionally reflect the integrated dietary intake of an individual, without substantial metabolic fractionation (28). This is because animal muscles are highly depleted in heavy zinc isotopes compared to other body tissues. Consequently, the consumer’s muscles will have lower zinc isotope ratios relative to their prey (25). In other words, Zn isotope ratios are a better proxy for vertebrate meat consumption than for trophic level. When combined with ecological proxies such as carbon and oxygen isotope data and trace element ratios (Ba/Ca and Sr/Ca) (see *SI Appendix*, Text 3 for a detailed description of isotopes and concentration ratios), δ^66^Zn becomes part of a robust multiproxy toolkit for reconstructing diet, ecology, and food access at the individual level.

By selecting dental enamel from both fauna and humans as the sole source of isotopic and trace-element data, our analysis primarily targets diet during early life while also capturing dietary patterns in later, postweaning phases, thereby providing valuable insight into feeding practices and broader food traditions.

We hypothesize that the diet, lifeways, and human–animal relationships at Abu Tbeirah were shaped by social status, as reflected in differential access to food resources and animal products.

From this hypothesis, three testable predictions derive:

i) If the diet was that of nonelite groups, it should be primarily based on C_3_ crops, with limited access to animal protein (20). Variability beyond expectations derived from textual models is expected, given their elite-focused nature.
ii) Given the location of the site and the overall archaeological record, high trophic aquatic resources are expected to have had a role in the diet of Abu Tbeirah inhabitants.
iii) If animal management followed nonelite household practices (29), species-specific isotopic signatures should reflect free-range herbivore feeding and pigs’ access to domestic by-products, rather than controlled C_3_-cereal based foddering associated with intensive practices aimed at higher quality products (23).

## Results

The zinc, oxygen, and carbon isotope values, along with trace element concentrations, are reported in *SI Appendix,* Table S7. Chemical alteration assessment is reported in *SI Appendix*, Text 3.

Statistical tests (Welch ANOVA with Games–Howell post hoc) were evaluated in conjunction with the width and overlap of 95% CI, following the approach proposed by Giaccari et al. (2024) and Vaiglova et al. (2023) (30, 31). Full procedures are described in *SI Appendix*, Text 5. Results of the statistical tests for isotopic values are summarized in *SI Appendix,* Tables S8 and S9 (complete dataset and outlier-removed), and linear correlations for trace elements in *SI Appendix,* Table S10.

### Zinc Isotope Ratios.

Zinc isotope values clearly distinguish trophic positions among faunal groups, with humans showing lower δ^66^Zn values than both herbivores and omnivores. Mammals’ zinc isotope values range from 0.58 to 1.58‰ (mean ± SD: 0.83 ± 0.23‰). Median ± MAD_norm_ (normalized median absolute deviation) values by dietary category are grazers (N = 5) 1.04 ± 0.15‰, omnivores (N = 6) 0.81 ± 0.04‰, sheep/goats (N = 6) 1.27 ± 0.01‰, and humans (N = 31) 0.69 ± 0.09‰. This trophic differentiation mirrors patterns observed in modern and prehistoric food webs (25, 32) with the notable exception of grazers, which present depleted values.

Statistical analyses (*SI Appendix,* Tables S8 and S9 and Text 5) reveal no significant difference in the means between omnivores and grazers (a result influenced by an outlier in the *Sus* group), and statistically significant differences between omnivores and sheep/goats. The two herbivore groups do not differ significantly from each other. Humans show statistically significant differences from all dietary groups other than grazers, with the latter pattern also affected by outliers.

### Oxygen and Carbon Isotope Ratios.

Oxygen and carbon isotopes discriminate between *Gastropoda* (marine), freshwater, and terrestrial taxa, and further differentiate terrestrial animals into herbivores and omnivores.

Human carbon isotopes suggest a C_3_ plant-based diet (converted dietary median ≈−23.5‰, following the corrections proposed by Passey et al. (33), *SI Appendix*, Text 4.1).

Median ± MAD_norm_ δ^18^O values by dietary category are grazers (N = 5) −0.72 ± 1.02‰, omnivores (N = 6) 0.03 ± 0.88‰, sheep/goats (N = 6) −0.57 ± 0.47‰, and humans (N = 29) −4.18 ± 0.99‰.

Median ± MAD_norm_. δ^13^C values by dietary category are grazers (N = 5) −4.80 ± 2.11‰, omnivores (N = 6) −9.53 ± 1.52‰, sheep/goats (N = 6) at −6.13 ± 0.73‰, and humans (N = 29) −11.56 ± 0.50‰.

As expected, statistical analyses (*SI Appendix*, Text 5) on δ^18^O, when excluding the outliers, reveal significant differences in the means between humans and all faunal groups and among distinct faunal groups. Interestingly, no significant differences were found between obligate and nonobligate drinkers.

Similarly, the analysis of δ^13^C values showed statistically significant differences across most groups, except grazers vs. sheep/goats and humans vs. omnivores.

### Trace Element Ratios (Ba/Ca and Sr/Ca).

Trace-element concentrations measured in both humans and animals provide an additional line of evidence supporting differences in plant and animal product consumption. The log(Ba/Ca) ratios range from −5.44 to −3.29 (N = 55), while log(Sr/Ca) ratios range from −3.12 to −2.00 (N = 55). As expected, grazers exhibit higher Ba/Ca and Sr/Ca values than carnivores and browsers (*SI Appendix,* Table S7) (34). Sheep/goat specimens show elevated ratios relative to grazers, followed by omnivores and humans. Humans display the lowest Ba/Ca and Sr/Ca ratios, indicating distinct dietary inputs and potentially higher intake of animal-derived protein (*SI Appendix*, Fig. S9).

Considering linear correlations, both Ba/Ca and Sr/Ca ratios covary strongly with carbon isotope values, consistent with their role as proxies for distinguishing between plant and meat intake.

Both Ba/Ca and Sr/Ca ratios also correlate with zinc isotope values. However, this relationship becomes negligible when restricting the analysis to postweaning individuals, suggesting that the original correlation may have been driven primarily by breastfeeding-related physiological signals rather than dietary intake alone.

## Discussion

Each proxy (δ^13^C, δ^18^O, δ^66^Zn) reflects distinct physiological and ecological processes during enamel formation, with differing temporal and metabolic sensitivities.

Their combined interpretation allows us to test five predictions regarding diet, early-life nutrition, and animal management at Abu Tbeirah.

The context allows us to explore the interaction between household-level provisioning and broader institutional food redistribution, without formally testing socioeconomic models. To account for the limited availability of published zinc isotope data, comparisons included sites spanning broad temporal and geographic ranges (*SI Appendix*, Text 4.4).

### Trophic Dynamics and Animal Management at Abu Tbeirah.

**Fig. 2.**
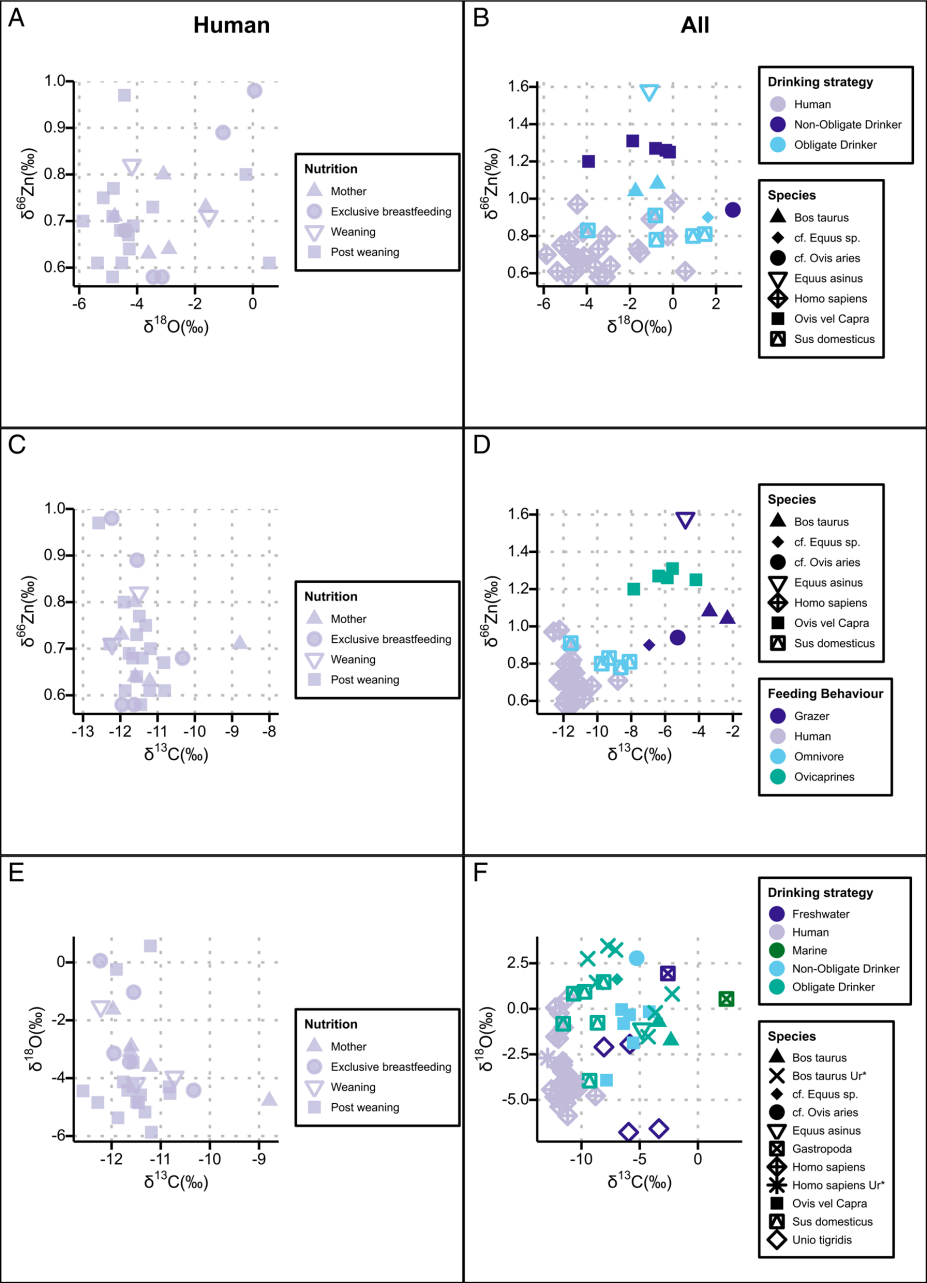
Scatterplots of isotopic values divided in (*A*) δ^66^Zn vs. δ^18^O in humans, grouped by feeding behavior; (*B*) δ^66^Zn vs δ^18^O, with symbols indicating species and color indicating drinking strategy; (*C*) δ^66^Zn vs. δ^13^C in humans, grouped by feeding behavior; (*D*) δ^66^Zn vs. δ^13^C, with symbols indicating species and color indicating feeding behavior; (*E*) δ^18^O vs. δ^13^C in humans, grouped by feeding behavior; (*F*) δ^18^O vs. δ^13^C, with symbols indicating species and color indicating drinking strategy, compared with available data from Ur: *Bos taurus* (36), Humans (37, 38); for humans Nutrition is a simplified version of the one presented in *SI Appendix,* Table S1.

We first test whether animal management at Abu Tbeirah reflects low-intensity, household-based practices or more centralized practices, using δ^66^Zn and other isotopic proxies. The failure of collagen preservation at Abu Tbeirah (*SI Appendix*, Text 1) makes zinc isotopes an essential proxy for reconstructing consumption of meat and animal products in southern Mesopotamia. As expected, herbivores at Abu Tbeirah exhibit higher δ^66^Zn values than omnivores, with the trophic level spacing between humans and herbivores (TLS_humans-herbivores_) of +0.45‰ (Fig. 2*A*), consistent with the modern baseline of Koobi Fora (Kenya, 0.44‰, excluding bone-eating carnivores) (25) and previous observations (e.g., refs. 27 and 35).

Additionally, as observed in open landscapes (25, 27, 32, 35, 39) but not in tropical rainforests (40, 41), there is a clear distinction between grazers and undifferentiated sheep/goats, likely representing either sheep (*Ovis aries*, grazers) or goats (*Capra hircus*, browsers). This distinction may reflect the higher δ^66^Zn values typically associated with cereals compared to other plant types. At Abu Tbeirah, the elevated δ^66^Zn values among undifferentiated sheep/goats may tentatively point to *Capra hircus* (goat), with a possible contribution from Ca-rich, ^66^Zn-enriched browsed plants. This may offer a glimpse into the herding and subsistence strategies of the population: In Mesopotamia, such animals were generally kept for subsistence rather than for market purposes (42). Alternatively, this isotopic offset could reflect environmental or dietary differences not captured by carbon or oxygen isotope data (Fig. 2 *B*, *D*, and *F*). Another plausible explanation is that these sheep/goats followed different feeding regimes than other browsers, as also suggested by their Ba/Ca and Sr/Ca ratios (*SI Appendix*, Fig. S9).

The extremely high values of the buried donkey (1.58‰) support a hypergrazer behavior (39) different from what is observed in the other equid of the analyzed sample, whose values reflect a more mixed diet; this in turn may suggest that such equid might belong to a different species (i.e., *Equus hemionus*), to a hybrid or that it was fed differently or in a different bedrock from the buried donkey.

The clear trophic differentiation (Fig. 2 *B*, *D*, and *F*) between feeding groups makes zinc isotopes not only a powerful tool for reconstructing local ecology and niche partitioning but also for understanding aspects of human behavior. Pigs show values consistent with an omnivorous diet, suggesting that they were fed on human food scraps and scavenged within the settlement, possibly consuming C_3_ plants like reed. Their management was likely household-based, rather than institutional (43). These animals were, in fact, well-suited to live in a small plot of land and were not taxed by institutions (43).

The relatively high carbon isotope values (Fig. 2 *D* and *F*) indicate that herbivores consumed a mixture of C_3_ and C_4_ plants, with *Bos taurus* showing a strong reliance on C_4_ plants (*SI Appendix*, Fig. S9). It is plausible that animals grazed on vegetation from the margins of irrigation canals, where water stress and soil salinity, known to enrich isotopic values (44), were elevated. Alternatively, they may have fed in marshland environments, where dominant plants follow the C_4_ photosynthetic pathway. A comparison of *Bos taurus* specimens from Ur and Abu Tbeirah reveals that some cattle shared similar foddering environments across both cities, while others were likely fed only with C_3_ plants, similar to the pigs and *Ovis aries*. This suggests at least two distinct foddering regimes. Such differentiated herding strategies stand in contrast to the pattern observed at the northern Syrian site of Tell Tweini (45), where all domestic animals (with no differentiation according to species) consumed predominantly C_3_ diets.

The isotopic differentiation among herbivores and the feeding regime of pigs is consistent with low-intensity, household-level management practices and does not support intensive exploitation.

These ecological baselines and feeding behaviors provide a critical framework for interpreting the human zinc isotope values at Abu Tbeirah, linking broader aspects of animal management, subsistence strategies, and social organization within the community.

### Human Data: Food Access and Childhood Nutrition.

Next, we evaluate diet composition, interindividual variability, and the contribution of aquatic resources in light of expectations derived from social status and local ecology.

Zinc isotope ratios indicate a predominantly omnivorous diet, although some individuals exhibit slightly more elevated trophic levels. The relatively high trophic level spacing between humans and herbivores (TLS_humans-herbivores_) of 0.45‰ points to an intermediate trophic position. When considering the TLS_humans-omnivores_ (0.12‰), the data suggest similar trophic positions within this group. However, although pig meat may have contributed to the diet, as suggested by other isotopic proxies, their consumption was likely not extensive, with cereals providing a proportionally greater contribution. A relatively low intake of animal protein is consistent with the social status of the individuals represented in this assemblage.

Abu Tbeirah’s TLS_humans-herbivores_ is comparable to that reported at Taforalt (+0.34‰, Morocco), where a high reliance on cereal-based diets was confirmed by compound-specific isotope analysis of amino acids (CSIA-AA) (27). In contrast, the historical population of Rennes (France) exhibited a higher offset (+0.63‰), reflecting greater reliance on meat and fish consumption, consistent with the archaeological context (46).

Carbon isotope and trace element data suggest that pigs were the primary source of meat (Fig. 2*D* and *SI Appendix*, Fig. S9), with smaller contributions from sheep/goats or their secondary products. The relatively narrow difference in values between humans and pigs is consistent with expectations for a nonelite diet with limited access to animal protein.

**Fig. 3.**
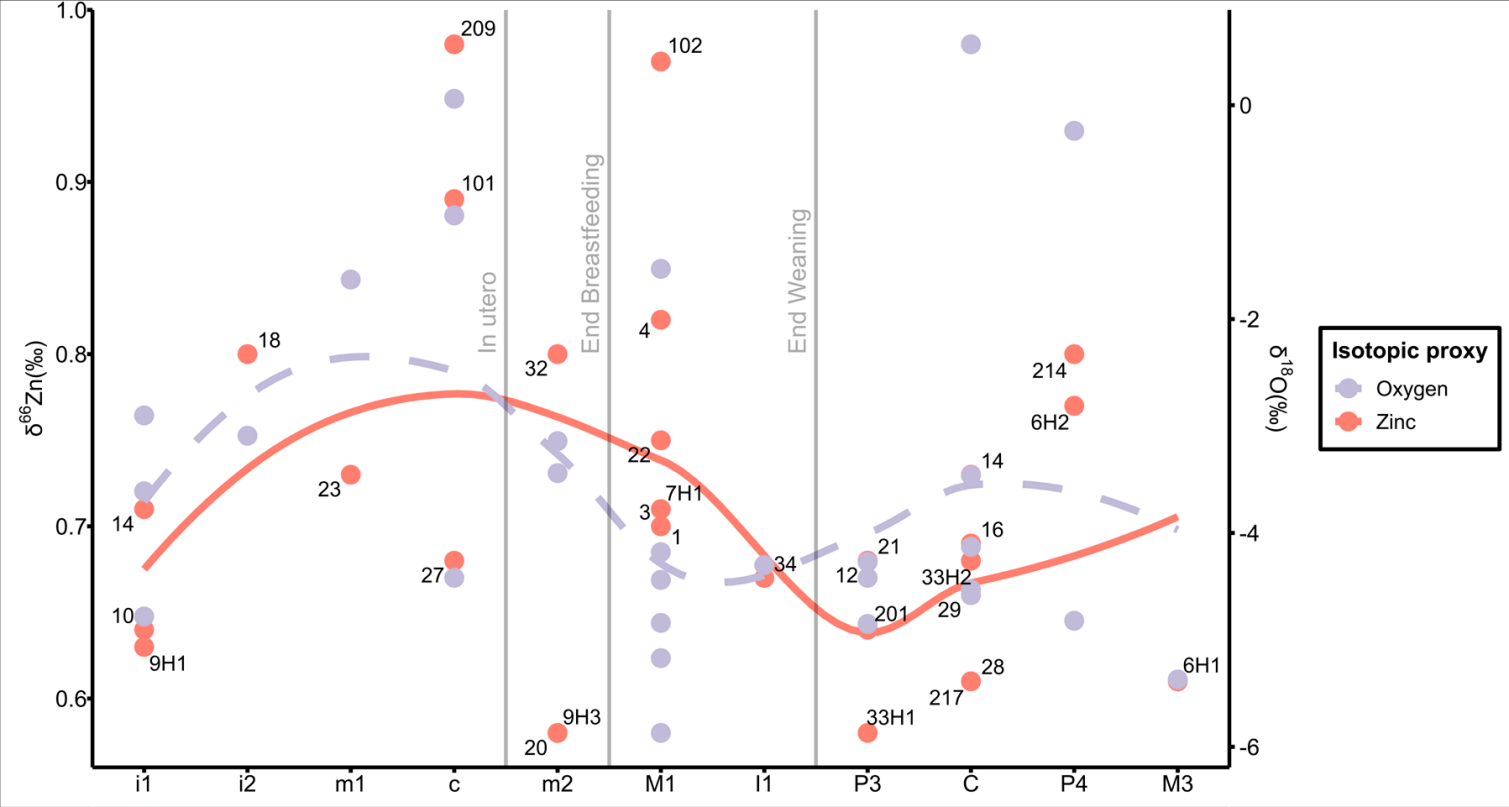
Zinc and oxygen isotope values are ordered by tooth formation sequence. Colors indicate the isotope value. Vertical gray bands mark broad developmental phases used to investigate weaning and breastfeeding. The curves represent a LOESS smoothing of δ^66^Zn (solid line) and δ^18^O (dashed line) values across the sequence of sampled teeth.

When focusing exclusively on postweaning individuals, so as to have a better understanding of early diet, intrapopulation variability in zinc isotopes decreases to approximately +0.2‰, as shown in Fig. 3. Comparison of the δ^66^Zn dispersion (IQR) with published data reveals a similarly homogeneous dietary pattern to the one observed at the Iberomaurusian site of Taforalt and the middle Holocene site of Lapa do Santo (*SI Appendix,* Table S11 and Text 4.4), where the group likely had homogeneous access to resources as opposed to urbanized societies. Additionally, when investigating the Interdecile Index (IDI), a proxy of dietary inequalities among human populations (Colleter et al., under revision), similarities emerge with the craftsman medieval population of Saint Laurent and the Iberomaurusian site of Taforalt (*SI Appendix*, Fig. S11).

In contrast, when including all individuals, variation increases to around 0.4‰, likely reflecting the influence of milk consumption during early tooth formation before weaning completion. Interestingly, oxygen isotopes mirror the trend of zinc isotopes values (Fig. 3), confirming variation in early diet and influence of milk consumption in isotopic values (47). The shift in the apex shape of the curve between oxygen and zinc isotopes can be partially explained by differences in tissue turnover. Zinc is incorporated during the enamel maturation phase, with an effective turnover on the order of 3 mo (48), thereby averaging isotopic inputs over a relatively broad period. In contrast, oxygen isotopes in enamel water exchange turn over much more rapidly [on the scale of days to weeks; (49)], yielding a signal that reflects a more temporally precise window. Zinc and oxygen isotopes also reveal new insights into early-life feeding practices, including breastfeeding duration, weaning transitions, and dietary supplementation in infancy. Isotopic research on breastfeeding and weaning in Mesopotamia remains limited due to poor collagen preservation, even in dentine, prohibiting, for example, incremental studies. Stantis and colleagues (15) successfully analyzed two Mesopotamian areas, estimating an exclusive breastfeeding period between 0.4 and 0.5 y, with weaning concluding between 2.3 and 3.1 y. These estimates align well with ethnographic and ethnohistorical data from preindustrial societies (50) and cuneiform texts referencing wet nurses, whose contracts generally lasted 3 y (51). Additionally, as these contracts were subscribed by higher-class women to lower-class ones, the latter would have benefited from shorter birth intervals (52).

Enrichment in zinc isotope values is evident in teeth that form at a time potentially recording the transition from in utero (mother) nutrition to exclusive breastfeeding. In Figs. 2*A* and 3, individuals from graves 209 and 101 clearly reflect this pattern, considering that the tooth used (a deciduous canine) forms at such a transition. Similarly, individuals from grave 4, for which we analyzed a first permanent molar, show enriched zinc values coupled with enriched oxygen values, whereas the one from grave 102 shows a greater cereal consumption. They appear to be in the weaning phase and may have supplemented their diet with animal milk and cereals, a common practice among the Sumerians (53). Animal milk, particularly cow’s milk, is enriched in δ^18^O by 2 to 6‰ (54), accounting for the elevated oxygen isotope values observed. Notably, as highlighted by Jaouen and colleagues (55), teeth less affected by breastfeeding signals, such as incisors, premolars, canines, and third molars (*Materials and Methods*), show lower zinc isotope values. Zinc and oxygen isotope data together allow us to reconstruct nuanced transitions in infant feeding strategies, despite the absence of preserved collagen or direct textual accounts.

When considering the complete dataset, fish, particularly marine species, appear to have played a minimal role in the diet (Fig. 2 *B* and *D*). Nonetheless, given the carbon and oxygen isotope values of humans in Abu Tbeirah, the occasional consumption of freshwater fish and mollusks remains plausible for some individuals (56) (Fig. 2*F*). This pattern is unexpected given the position of the site, and the archaeological and textual evidence suggesting a strong relation between the population and the riverine/marshland system. Critically, the consumption of shellfish (a low-trophic species) may be isotopically difficult to detect and particularly when consumed in small quantities. Although marine fish are underrepresented archaeologically, they feature in textual sources as an important dietary staple in southern Mesopotamia for both lower- and higher-class individuals (24, 57). The lack of isotopic evidence for marine food consumption at Abu Tbeirah invites a reevaluation of their dietary role in coastal Mesopotamia. These results suggest that not only the actual availability and the presence of remains in the archaeological record shaped food preferences but also other factors, such as the exchange value of high-trophic fish, cultural practices, and taste, also played a role in determining what was actually consumed.

Consistent with this interpretation, collagen-based studies on coeval sites such as Sidon (on the Mediterranean coast), with which the Mesopotamians had connections in later periods, similarly point to a minor contribution of marine resources (58).

Beyond general dietary patterns, this multiproxy approach also reveals subtle forms of dietary differentiation within the community, possibly reflecting access to food, status, or labor roles.

Two individuals (graves 214, 6H2) exhibit elevated zinc isotope values despite representing a postweaning diet. Interestingly, for the individual from grave 214, zinc values correlate positively with oxygen isotope data (Fig. 2*A*), which might tentatively indicate continued animal secondary products consumption. This does not appear to happen for the individual from grave 6H2 whose diet seems to suggest a high reliance on plants. However, it should be noted that this individual has high Al and Fe concentrations, possibly suggestive of contamination.

Individuals from graves 214 and 217 were recovered from the area of an administrative building (Area 6), dated to a later, more arid period, a context also associated with the presence of higher social status individuals [*SI Appendix*, Text 2.4, (59)]. These individuals may have had access to higher-quality foodstuffs (60), potentially explaining their zinc isotope data. The enriched values for individual 214 might be explained through their consumption of secondary products, which notably produced higher zinc ratios. Individual 217, who plots in the lower section of the diagram, might have instead consumed meat, which translates into low zinc values.

Heterogeneous values are also shown by individual 6H1 and 6H2. Their differences in zinc data might call for an interesting food complexity even between individuals that could have had a clear bond during their life (or at least one that justified a communal burial; See *SI Appendix*, Text 2.2). The male individual (6H2), found in secondary deposition, shows signs of intense physical labor and is associated with low nitrogen values ((12) and *SI Appendix*, Text 2.5), which seem to match the high zinc values, suggesting a plant-rich diet. The primary deposition of the female (6H1), with low zinc values, suggests a meat-rich diet, although no nitrogen values are available to support this hypothesis (Fig. 2 *A* and *C* and *SI Appendix*, Fig. S9). The greater intrapopulation variability observed in certain cases is consistent with what was expected from the elite-biased ration texts, suggesting that they may not fully represent actual food practices.

## Conclusions

Zinc isotope analysis of dental enamel provides a viable alternative for reconstructing diet in southern Mesopotamia, where collagen preservation is poor. Zinc and carbon isotope values, together with trace element ratios, indicate a C_3_ cereal-based omnivorous diet with limited, though not absent, access to animal proteins, primarily from meat, namely of pigs. This pattern is consistent with the dietary expectations for a largely nonelite urban population. Zinc and oxygen isotope data further clarify breastfeeding and weaning trajectories, offering a fine-grained view of early-life nutrition.

Isotopic evidence indicates that high-trophic marine resources did not play a consistent role in the diet of the individuals analyzed. Carbon and oxygen isotopes, along with trace element ratios, further indicate that freshwater intake played at most a minor role.

Isotopic variation in the fauna reveals flexible, low-intensity management practices: Pigs likely fed on household by-products while herbivores were managed in free-range systems. This pattern reflects household-level provisioning rather than centralized or intensive management strategies.

Overall, these results support the idea that social status shaped diet and access to animal products at Abu Tbeirah, while also paving the way for broader application of zinc isotopes in arid and saline environments globally, where collagen preservation has historically limited bioarcheological research. The isotope data complement historical sources by identifying which species were most frequently consumed and showing that fish made, at most, a minor contribution to nonelites’ diets. Future studies should adopt this approach at other Mesopotamian sites and supplement existing isotope data to clarify dietary patterns, animal management, and social organization more clearly during the third millennium BCE.

## Materials and Methods

### Materials.

A total of 33 human and 30 faunal specimens were selected at Abu Tbeirah between 2012 and 2019. Of these, 31 human and 18 animal enamel samples were analyzed for zinc isotopes and trace elements. The faunal sample set includes 11 herbivores divided into five grazers (2 *Bos taurus*, 1 *Equus* sp., 1 *Equus asinus*, 1 cf. *Ovis aries*) and six ovicaprines (sheep/goats; *Ovis* vel *Capra*), six omnivores (5 *Sus domesticus*, 1 *Sus scrofa*), and one fish.

Trace element (TE) analysis was performed on the same mammals and fish, with the addition of three *Unio tigridis* and two gastropods.

An additional sample of 29 human and 24 faunal specimens were analyzed for carbon and oxygen isotopes.

### Tooth Sampling Strategy and Life History Stages.

All isotopic and elemental analyses (δ^66^Zn, δ^13^C, δ^18^O, Ba/Ca, and Sr/Ca) were conducted on dental enamel, a highly mineralized tissue that forms during childhood and does not remodel. As a result, all biogenic signals reflect dietary and environmental conditions at the time of tooth formation and remain stable throughout the individual’s life. To address early-life dietary variation, human enamel was sampled in accordance with known patterns of tooth mineralization. For permanent teeth, enamel was removed at three-quarters of crown height near the enamel–root junction (ERJ); for deciduous teeth, sampling focused near the cusps. Based on formation stages (61), teeth were broadly grouped into the following life-history phases: in utero (expressing mother values; di1, di2, dm1), Exclusive Breastfeeding (dc, dm2), weaning (M1), and postweaning (I1, P3, C, P4, M3). Breastfeeding leaves a recognizable isotopic signature, typically elevating δ^18^O by +2 to 3‰, δ^13^C by +1‰ (47), and increasing δ^66^Zn values (55). Trace element ratios also reflect dietary stage: Ba/Ca rises with breastfeeding, while Sr/Ca declines, both remaining low in utero (62).

### Assessment of Diagenesis and Preservation.

Although it is well attested that dental enamel preserves biogenic Zn, we confirmed that most of the samples were not altered by diagenetic processes. We analyzed elements abundant in soil (Fe, Al, Mn) but not abundant in dental enamel. Finally, it is of great importance to compare the Zn isotopic ratio with the Zn concentration of multiple species from the same site (63). Previous studies have shown no correlation between the values in dental enamel (26, 41, 64). The only sample excluded from the discussion is the fish, whose values were likely contaminated.

### Methods.

All labware, including new materials, was cleaned to eliminate exogenous zinc contamination. Cleaning procedures were performed using nitrile gloves worn beneath vinyl gloves to minimize Zn exposure (55).

Zn isotope compositions were measured following the protocols by Moynier et al. (65) and modified by Jaouen et al. (25). Enamel samples from humans (5.4 to 19.6 mg) and animals (5.3 to 27 mg) were digested in 1 mL of 1 N HCl at 100 °C, evaporated to dryness, and redissolved in 1 mL of 1.5 N HBr. A 0.05 mL aliquot was extracted for trace element analysis.

Ion exchange chromatography was performed using 1 mL AG-1X8 resin (200 to 400 mesh). Columns were conditioned with 3 mL of 1.5 N HBr. The sample matrix was eluted with 2 mL of 1.5 N HBr, and Zn was eluted with 5.5 mL of 0.5 N HNO_3_. This purification cycle was repeated twice.

Isotopic measurements were carried out on a Thermo Neptune MC-ICP-MS at the *Geoscience Environnement Toulouse* (GET) following the protocol of Toutain and colleagues (66) and using Cu doping. The measurement of Cu doping (known isotopic values) along with Zn is needed to correct the instrumental fractionations caused by the MC-ICPMS. A subset of samples was measured in duplicate or triplicate.

Care was taken to prevent the formation of Br_2_ gas during purification, caused by residual nitrates reacting with HBr. Its presence (coloration of the sample) may result in isotope fractionation due to premature Zn elution, favoring lighter isotopes (35). To avoid this, we conducted a simple visual inspection.

Aliquots for trace elements measurement were analyzed with a triple quadrupole ICP-MS (iCapTQ, Thermoscientific) (*Observatoire Midi Pyrénées* OMP, Toulouse, France). A calibration line was established to fit the range of the concentration observed in teeth.

Stable carbon and oxygen isotopes were measured at former Iso-Analytical (now Sercon Analytical) using a Continuous Flow-Isotope Ratio Mass Spectrometry (CF-IRMS). Between 5.1 and 17.7 mg of human enamel and between 5.7 and 47.7 mg of carbonate shell or animal enamel were sampled. Samples were flushed with 99.995% pure helium gas. Consequently, H_3_PO_4_ was added and left overnight to convert all the samples into CO_2_. The gas was sampled into a continuous flow of He and forwarded to a Europa Scientific 20-20 IRMS, where it was ionized, separated in relation to mass, and measured in a Faraday cup.

Boxplots were used to identify potential outliers, defined as values outside 1.5× the interquartile range (IQR, *SI Appendix*, Fig. S10). Additionally, to balance accessibility and statistical rigor, group comparisons were interpreted using a combination of hypothesis testing and 95% CI. In line with best practice, nonoverlapping CIs were taken as conservative evidence of a statistically significant difference, as detailed in *SI Appendix*, Text 5. All test types, distribution checks, and comparisons are fully reported in *SI Appendix,* Tables S8 and S9 and Text 5. This approach enables transparent interpretation of dietary variability while avoiding overfitting the limited number of categorical comparisons.

## Supplementary Material

Appendix 01 (PDF)

## Data Availability

R script data have been deposited in Zenodo (67). Other data are included in the article and/or *SI Appendix*.
