# Supplementary material for: When collagen fails: Zinc isotopes unlock Sumerian lifeways in southern Mesopotamia
Source: Proc Natl Acad Sci U S A. 2026 Mar 9;123(11):e2526276123. doi: 10.1073/pnas.2526276123 (PMC12993939; doi:10.1073/pnas.2526276123)
Supplement: Supplementary file 1 — Appendix 01 (PDF) [file pnas.2526276123.sapp.pdf]

PNAS

Supporting Information for
When Collagen Fails: Zinc Isotopes Unlock Sumerian Lifeways in Southern Mesopotamia

Matteo Giaccari*, Licia Romano, Silvia Soncin, Sofia Panella, Francesca Alhaique, Franco D'Agostino, Klervia Jaouen, Mary Anne Tafuri

*Email: matteo.giaccari@uniroma1.it; giaccari98@gmail.com

This PDF file includes:

Supporting text 1 to 5
Figures S1 to S11
Tables S1 to S11
SI References

Supplementary Text 1

Historical and Archaeobotanical Context of Diet and Animal Management in Southern Mesopotamia

Cuneiform texts are primarily administrative and elite-focused, however, they provide valuable insights into diet and animal use, particularly if associated with other indirect evidence.

Textual sources and archaeobotanical evidence suggest diets centered on C_3 cereals, particularly barley and wheat, along with secondary products like bread, beer, porridge, dairy, and sesame oil playing key dietary roles (1–7). Importantly, ration lists from the Early Dynastic period in Lagash commonly included bread, beer, and fish, as well as roasted and green barley (3, 8). Although marine fish are underrepresented archaeologically, they could have been an important dietary staple in southern Mesopotamia for both lower and higher class individuals (8, 9). Notably, the quantity of barley distributed varied according to the sex and age of the recipients. Even young children received regular rations that increased with age, suggesting that the quantity, rather than the quality, of food differed between adults and children. Moreover, wet nurses' contracts suggest that weaning did not occur until the second or third year of life (10). While such records are invaluable, they remain exceptional and do not provide direct insight into the actual foods consumed by individuals. However, given the limitations of textual sources in reconstructing non-elite diets, direct isotopic analysis remains essential.

Livestock exploitation patterns suggest differences in rural and urban contexts dictated by the value of their secondary products, with goats preferred for milk in villages and sheep for wool in cities (11). Pigs, raised primarily for meat, are underrepresented in administrative texts, though Old Sumerian documents attest that they were fed on a variety of products, including barley, bran, reeds and fresh dates (12) but also human leftovers (8). In the absence of isotopically analyzed reference taxa from the region, the reconstruction of herding strategies must rely heavily on textual, zooarchaeological, and ethnographic data. As outlined by Greenfield and colleagues (13), we can propose some possible herding strategies: foddering by reeds (a method still used in modern Iraq), fattening with barley, feeding livestock with household leftovers, pasturing animals at the distal ends of irrigation systems, adjacent to the steppe.

Supplementary Text 2
Abu Tbeirah: Site, Faunal assemblage, Environment and Previous Investigations

2.1 The city of Abu Tbeirah

Abu Tbeirah (30.813056, 46.678611, Main Text Figure 1, Figure S1) is a medium-sized Sumerian city (42ha) located 7 km south of the modern city of Nasiriyah in former southern Mesopotamia. The site comprises domestic areas, presumed institutional buildings, and a harbor structure (2). Its main occupation occurred during the Early Dynastic period (2900–2350 BCE), followed by a contraction northeastward to Area 6 during the Ur III and early Old Babylonian phases (early 2nd millennium BCE) (14, 15). Domestic architecture has been uncovered in the Areas 1, 2, and 4, with the first and the latter featuring larger buildings (14). Human and faunal remains were recovered from all areas.

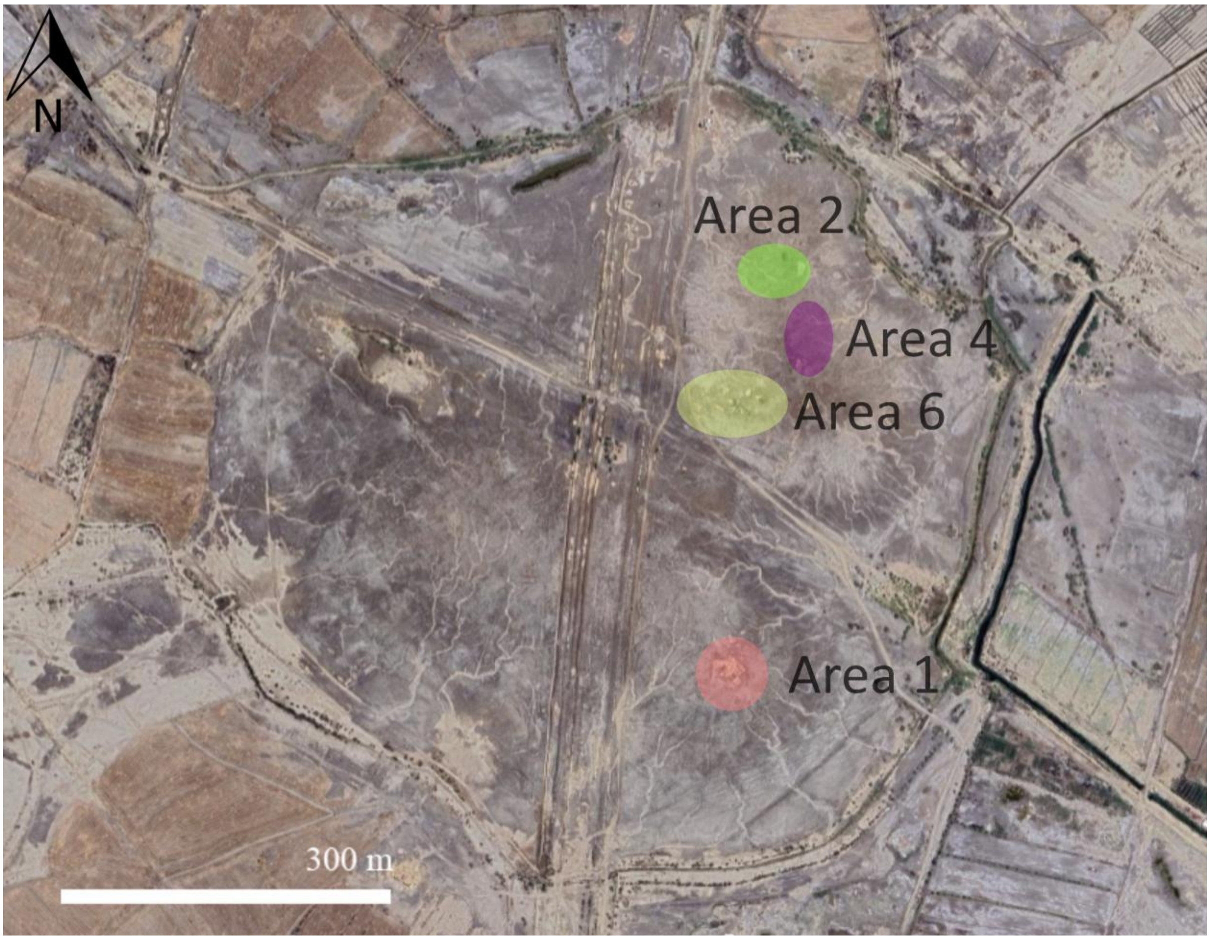

Figure S1. Plan of the site of Abu Tbeirah showing the main excavated areas (2). Modified from ©2024 Google Earth from Giaccari et al., forthcoming

2.2 Human recovered at Abu Tbeirah

Human individuals recovered at Abu Tbeirah with teeth available for isotopic analysis are summarized in Table S1. Most of the individuals included in this study derive from domestic or cemeterial contexts, characterized by the absence of monumental architecture and prestigious grave goods. This pattern, well documented in the archaeological synthesis of the site (2), identifies the individuals recovered as members of the ordinary, non-elite urban population. A small number of atypical burials from Area 6 with distinct markers are discussed elsewhere (14).

It is worth noting the direct association of the individuals from grave 6. In detail, the secondary deposition of 6H2 is placed over the lower limbs of the primary burial of 6H1; moreover, both individuals share a morphological variant (septal aperture of the olecranon) (2). These observations, recorded during the excavation and the analysis of the remains, suggest that they were intentionally buried in close association. Nonetheless, no formal inference of biological relatedness is intended.

2.3 Animal Management at Abu Tbeirah

The faunal assemblage is dominated by ovicaprines and pig, with lower frequencies of fish and mollusks, and only rare occurrences of cattle remains (Figure S2, data from 16, 17). The unusually high frequency of *Canis familiaris* mainly reflects the presence of a dog burial in Room 22 (16–18), while the apparent abundance of fish outside funerary contexts results mostly from a concentration of fish remains contained in a large dish from Room 1, Building A (16, 19).

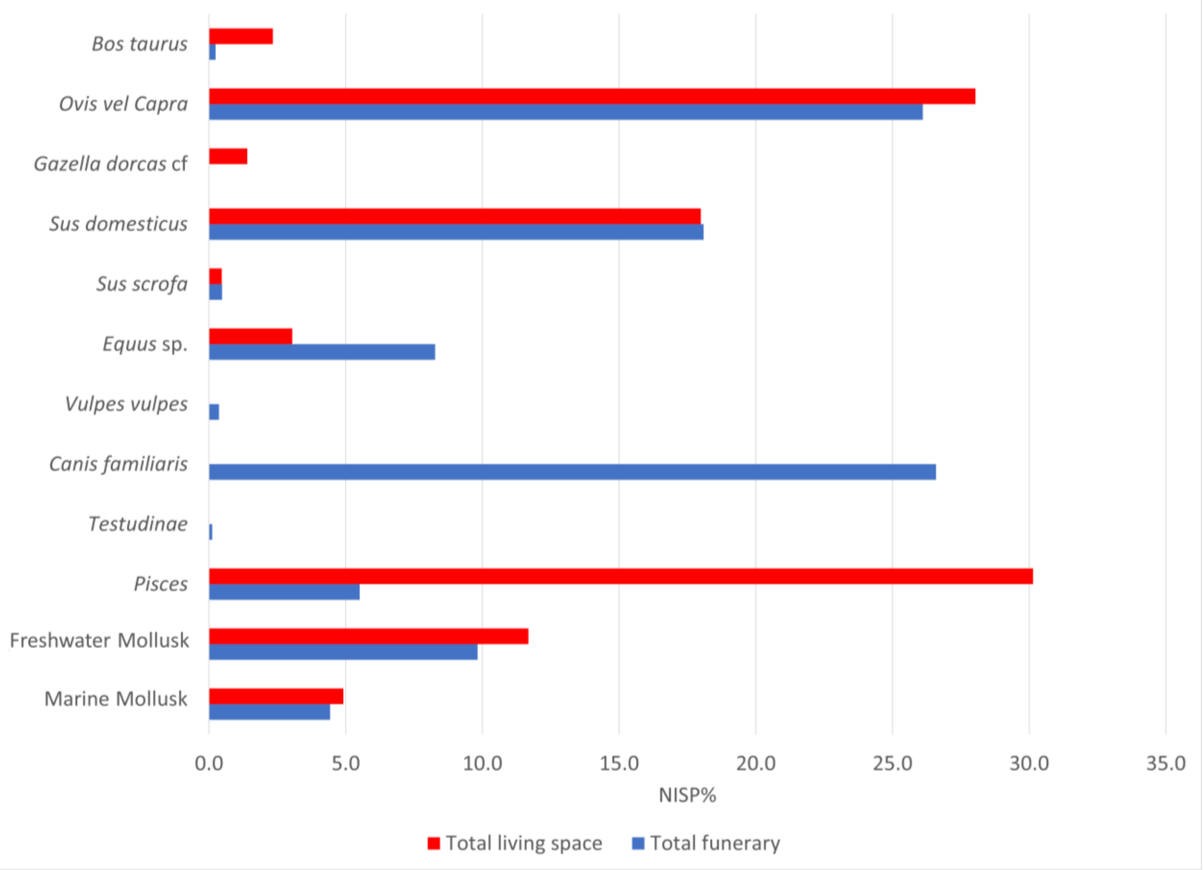

Figure S2. Frequency of different taxa in the Abu Tbeirah faunal assemblage divided by type of context (funerary vs. living; NISP = Number of Identified Specimens)

The relative prominence of pig remains, apparently at odds with cuneiform sources, is likely due to their household-level management, which would have excluded them from administrative records (12, 17). Morphometric analysis (16) and forthcoming isotope data, suggest that pigs were raised within the city perimeter, reducing interaction with wild boar. Additionally, although bird remains are almost absent from the faunal assemblage (16), the presence of eggshell fragments in domestic contexts (20) suggests that eggs may have been part of the local diet.

2.4 Ecology at Abu Tbeirah

Abu Tbeirah occupied an ecotonal region between permanently flooded marshlands, dry marshlands, and adjacent steppe zones (21–27). Its occupation coincided with a major climatic shift: from a humid phase (ca. 4000–3000 BCE), which promoted deltaic progradation and floodplain irrigation (2, 28), to a progressive aridification (ca. 3000–2000 BCE), characterized by higher temperatures and increased evaporation rates (21, 29).
Vegetation in the permanently flooded marshes was dominated by C_3 plants, except for *Cyperus papyrus*, while drier marshes and steppe zones supported high densities of C_4 chenopods (13).

2.5 Previous stable isotope analysis

Between 2012 and 2017, 48 demineralized bone samples were analyzed for collagen preservation. Only 19 yielded collagen, of which only 3 met acceptable C:N ratios (30). These three samples, previously published by Tafuri (31), derived from 27 samples demineralised using HCl. Additional samples processed by S. Soncin, including 15 with EDTA and 6 with HCl, did not yield collagen. The three valid results suggest a C_3 cereal-based diet with low nitrogen isotope values ($\delta^{15}N$): Grave 3: (5.8‰), Grave 6H2 (2.5‰) and Grave 1 (7‰), the latter likely reflecting a breastfeeding signal. These $\delta^{15}N$ values are unusually low, especially considering the site's environmental and anthropogenic context. In theory, factors such as manuring, low precipitation, and marsh-dominated ecosystems tend to enrich, rather than deplete, $\delta^{15}N$ (1), making the observed results particularly notable.

Number	Area	SU	Context	Building	Room	Grave Goods	Grave	Age	Sex	Sampled element	species	Nutrition	Period	Feeding Behaviour	Drinking strategy
1	1	327	Cemeterial	Cemetery Area 1		pottery vessels	22	Adult	ND	ULM1	*Homo sapiens*	Probable Post weaning	ED/AKK	Omnivore (Human)	Obligate Drinker (Human)
2	2	590	Cemeterial	Cemetery Area 2		pottery vessels	102	Adult	F	URM1	*Homo sapiens*	Probable Post weaning	AKK	Omnivore (Human)	Obligate Drinker (Human)
3	1	186	Household	Area 1 Building A1	5	pottery vessels	14	Non-Adult	ND	LLC	*Homo sapiens*	Post weaning	ED/AKK	Omnivore (Human)	Obligate Drinker (Human)
4	1	652	Household	Area 1 Building A2	10	pottery vessels	34	Adult	ND	URI1	*Homo sapiens*	Post weaning	ED/AKK	Omnivore (Human)	Obligate Drinker (Human)
5	1	639	Household	Area 1 Building A2	17+19+21	No	32	Non-Adult	ND	URm2	*Homo sapiens*	Probable Exclusive Breastfeeding	ED/AKK	Omnivore (Human)	Obligate Drinker (Human)
6	1	114	Household	Area 1 Building A2	3	unknown	9 H3	Non-Adult	ND	URm2	*Homo sapiens*	Probable Exclusive Breastfeeding	ED/AKK	Omnivore (Human)	Obligate Drinker (Human)
7	1	186	Household	Area 1 Building A1	5	pottery vessels; Reed basket	14	Non-Adult	ND	i	*Homo sapiens*	Mother	ED/AKK	Omnivore (Human)	Obligate Drinker (Human)
8	1	276	Household	Area 1 Building A1	9	pottery vessels	20	Non-Adult	ND	LLm2	*Homo sapiens*	Probable Exclusive Breastfeeding	ED/AKK	Omnivore (Human)	Obligate Drinker (Human)
9	1	234	Household	Area 1 Building A2	6	pottery vessels	18	Non-Adult	ND	URi2	*Homo sapiens*	Mother	ED/AKK	Omnivore (Human)	Obligate Drinker (Human)
10	1	284	Cemeterial	Cemetery Area 1			21	Adult	ND	ULP3	*Homo sapiens*	Post weaning	ED/AKK	Omnivore (Human)	Obligate Drinker (Human)

11	1	475	Househ old	Building A2	10	No	29	Adult	F	LRC	*Homo sapiens*	Post weaning	ED/AKK	Omnivore (Human)	Obligate Drinker (Human)
12	1	467 0 462	Househ old	Building A2	22	pottery vessels, reed basket	28	Adult	M	LRC	*Homo sapiens*	Post weaning	ED/AKK	Omnivore (Human)	Obligate Drinker (Human)
13	6	166 7	Cemeter ial?	Area 6		unknown	217	Adult	F	URC	*Homo sapiens*	Post weaning	Ur III	Omnivore (Human)	Obligate Drinker (Human)
14	6	159 9	Cemeter ial?	Area 6		No	214	Adult	M	URP4	*Homo sapiens*	Post weaning	Old Bab	Omnivore (Human)	Obligate Drinker (Human)
15	1	449	Househ old	Building A2	22	pottery vessels, reed basket	27	Non-Adult	ND	LLc	*Homo sapiens*	Exclusive breastfeeding	ED/AKK	Omnivore (Human)	Obligate Drinker (Human)
16	6	153 8	Istitution al Househ old?	Area 6 Building E		pottery vessels, anklet in copper	209	Non-Adult	ND	ULc	*Homo sapiens*	Probable Exclusive Breastfeeding	Ur III	Omnivore (Human)	Obligate Drinker (Human)
17	4	104 6	Househ old	Area 4 Building D2	1	pottery vessels	201	Adult	M	LRP3	*Homo sapiens*	Post weaning	AKK	Omnivore (Human)	Obligate Drinker (Human)
18	1	649	Househ old	Area 1 Building A2	16	unknown	33H2	Adult	F	URC	*Homo sapiens*	Post weaning	ED/AKK	Omnivore (Human)	Obligate Drinker (Human)
19	1	643	Househ old	Area 1 Building A2	16	unknown	33H1	Adult	M	LRP3	*Homo sapiens*	Post weaning	ED/AKK	Omnivore (Human)	Obligate Drinker (Human)
20	1	367	Cemeter ial	Cemetery Area 1		pottery vessels, reed basket	23	Non-Adult	ND	Rm1	*Homo sapiens*	Mother	ED/AKK	Omnivore (Human)	Obligate Drinker (Human)
21	1	6	Cemeter ial	Cemetery Area 1		limestone spouted vessel, pottery vessels	1	Non-Adult	ND	LLM1	*Homo sapiens*	Probable Post weaning	ED/AKK	Omnivore (Human)	Obligate Drinker (Human)

22	2	574	Cemeterial	Cemetery Area 2		pottery vessels, Reed cradle	101	Non-Adult	ND	URc	*Homo sapiens*	Probable Exclusive Breastfeeding	AKK	Omnivore (Human)	Obligate Drinker (Human)
23	1	66	Cemeterial	Cemetery Area 1		No	6 H2	Adult	M	RP4	*Homo sapiens*	Post weaning	ED/AKK	Omnivore (Human)	Obligate Drinker (Human)
24	1	12	Cemeterial	Cemetery Area 1		pottery vessels	3	Adult	M	RM1	*Homo sapiens*	Weaning	ED/AKK	Omnivore (Human)	Obligate Drinker (Human)
25	1	43	Household	Building A1	outside	unknown	4	Non-Adult	ND	LM1	*Homo sapiens*	Weaning	ED/AKK	Omnivore (Human)	Obligate Drinker (Human)
26	1	55	Cemeterial	Cemetery Area 1		Organic Vessel, pottery vessel	6 H1	Adult	F	LM3	*Homo sapiens*	Post weaning	ED/AKK	Omnivore (Human)	Obligate Drinker (Human)
28	1	80	Household	Building A2	1	unknown	7 H1	Non-Adult	ND	LM1	*Homo sapiens*	Probable Post weaning	ED/AKK	Omnivore (Human)	Obligate Drinker (Human)
29	1	114	Household	Building A2	3	unknown	9 H1	Non-Adult	ND	i1	*Homo sapiens*	Mother	ED/AKK	Omnivore (Human)	Obligate Drinker (Human)
30	1	106	Household	Building A2	3	unknown	10	Non-Adult	ND	i1	*Homo sapiens*	Mother	ED/AKK	Omnivore (Human)	Obligate Drinker (Human)
31	1	145	Household	Building A1	4	pottery vessels, Reed basket, cosmetic shell	12	Adult	F	UP	*Homo sapiens*	Post weaning	ED/AKK	Omnivore (Human)	Obligate Drinker (Human)
32	1	197	Cemeterial	Cemetery Area 1		pottery vessels	16	Adult	M	LC	*Homo sapiens*	Post weaning	ED/AKK	Omnivore (Human)	Obligate Drinker (Human)
A1	1	53		Animal				Adult Animal		spine	*Pisces*	ND		Freshwater	Freshwater
A2	6	1603		Animal				Adult Animal		UM	*Bos taurus*	End lactation		Grazer	Obligate Drinker

A3	6	1637		Animal		Adult Animal	LP3	*Sus domesticus*	Post lactation	Omnivore	Obligate Drinker
A4	6	1577		Animal		Adult Animal	shell	*Unio tigridis*	ND	Freshwater	Freshwater
A6	6	1635		Animal		Adult Animal	shell	*Unio tigridis*	ND	Freshwater	Freshwater
A7	1		Cemeterial	Animal	20	Adult Animal	shell	*Unio tigridis*	ND	Freshwater	Freshwater
A8	6	1666		Animal		Adult Animal	M3	*Sus domesticus*	Post lactation	Omnivore	Obligate Drinker
A10	6	1577		Animal		Adult Animal	I3	*Ovis* vel *Capra*	Post lactation	Ovicaprines	Non-Obligate Drinker
A11	6	1571		Animal		Adult Animal	shell	Gastropoda	ND	Freshwater	Freshwater
A12	6	1601		Animal		Adult Animal	UP3/4	*Bos taurus*	Post lactation	Grazer	Obligate Drinker
A13	6		Cemeterial	Animal	212	Adult Animal		*Ovis* vel *Capra*	End lactation	Ovicaprines	Non-Obligate Drinker
A15	6	1613		Animal		Adult Animal	M2	*Sus domesticus*	Post lactation	Ovicaprines	Non-Obligate Drinker
A16	6	1639		Animal		Adult Animal	shell	Gastropoda	ND	Marine	Marine
A17	6	1578		Animal		Adult Animal	P4	*Ovis* vel *Capra*	Post lactation	Ovicaprines	Non-Obligate Drinker
A18	6		Cemeterial	Animal	214	Adult Animal	LD4	cf. *Ovis aries*	Mother	Grazer	Non-Obligate Drinker
A19	6	1562		Animal		Adult Animal	tooth	cf. *Equus* sp.	ND	Grazer	Obligate Drinker

A20	1		Cemeterial	Animal		21	Adult Animal	UI	cf. *Sus scrofa*	Post lactation	Omnivore	Obligate Drinker
A22	6	1566		Animal			Adult Animal	LP2	*Sus domesticus*	Post lactation	Omnivore	Obligate Drinker
A24	6	1639		Animal			Adult Animal	LP	*Sus domesticus*	Post lactation	Omnivore	Obligate Drinker
A26	1		Household	Animal		28	Adult Animal	UP2	*Sus domesticus*	Post lactation	Omnivore	Obligate Drinker
A27	2	557		Animal			Adult Animal	LM3	*Equus asinus*	Post lactation	Grazer	Obligate Drinker
A28	1			Animal		12 H1	Adult Animal		*Ovis* vel *Capra*	ND	Ovicaprines	Non-Obligate Drinker
A29	1	132	Cemeterial	Animal		11	Adult Animal	UM2	*Ovis* vel *Capra*	Post lactation	Ovicaprines	Non-Obligate Drinker
A31	1		Cemeterial	Animal		15	Adult Animal	Up4	*Sus domesticus*	Mother	Omnivore	Obligate Drinker

Table S1. Samples selected for Zn isotope and trace element analysis, including contextual information (SU = Stratigraphic Unit, ND = Not Determined, ED = Early Dynastic, AKK = Akkadian, Old Bab = Old Babylon, UL = Upper Left, UR = Upper Right, LL = Lower Left, LR = Lower Right, L = Lower, U = Upper; concerning teeth Uppercase is used for permanent dentition and lowercase for deciduous)

Supplementary Text 3
Diagenesis assessment

Due to the overall poor collagen preservation at Abu Tbeirah (31), dental enamel was selected as the primary substrate for isotopic analysis. Enamel is highly resistant to post-depositional alteration (32), making it suitable for preserving biogenic signals. Given the depositional context, several approaches were employed to assess the potential impact of diagenesis:

1. Comparison of elemental concentrations of exogenous soil-derived elements (Al, Mn, Fe) with known baseline values for uncontaminated enamel.
2. Evaluation of correlations between elemental concentrations and isotopic values, which could indicate mixing trends with a diagenetic endmember (33).
3. Assessment of enamel carbonate content (%CO_3).
4. Evaluation of Zn concentrations in contaminated faunal samples (particularly freshwater shells with inherently low Zn content).

3.1 Concentrations of Al, Mn, and Fe

Table S2 presents the concentrations of key trace elements used to identify potential diagenetic contamination (34). Several samples show elevated levels of Mn, Al, and/or Fe, notably:

- Individual 5 and the Pisces sample, which exceed thresholds for all three elements, suggesting exogenous contamination.

- Additional concern is noted for Individual 23, and faunal samples A18 (*Ovis aries*), A20 (*Sus scrofa*), A29 (*Ovis* vel *Capra*), and A31 (*Sus domesticus*), all of which exceed Mn and/or Fe thresholds.

- Individuals 10 and 24 show elevated Mn only, while sample 21 shows elevated Fe.

3.2 Correlation Between Zn Isotopes and Concentration of Potential Contaminants

Zn isotope values and concentrations display a modest but statistically significant correlation (Multiple R^2: 0.2416, Adjusted R^2: 0.2254, F-statistic: 14.97 on 1 and 47 DF, p-value: 0.0003). After excluding samples with elevated Al, Mn, and Fe, the correlation remains consistent (Multiple R^2: 0.2260, Adjusted R^2: 0.2045, F-statistic: 10.514 on 1 and 36 DF, p-value: 0.0026).
No significant correlation was observed between δ^{66}Zn and Fe (Multiple R^2: <0.0001, Adjusted R^2: -0.0213, F-statistic: <0.001 on 1 and 47 DF, p-value: 0.98932), and only a weak correlation with Mn (Multiple R^2: 0.1094, Adjusted R^2: 0.0904, F-statistic: 5.771 on 1 and 47 DF, p-value: 0.02029). Additionally, no correlation was observed with Al concentrations (Multiple R^2: 0.0428, Adjusted R^2: 0.0225, F-statistic: 2.104 on 1 and 47 DF, p-value: 0.15359), ruling out systematic detrital contamination. Comparable results are observed in the Spearman correlation heatmap (Figure S3).
These results suggest that the observed correlation between δ^{66}Zn and Zn concentrations is unlikely to be driven by diagenetic processes.

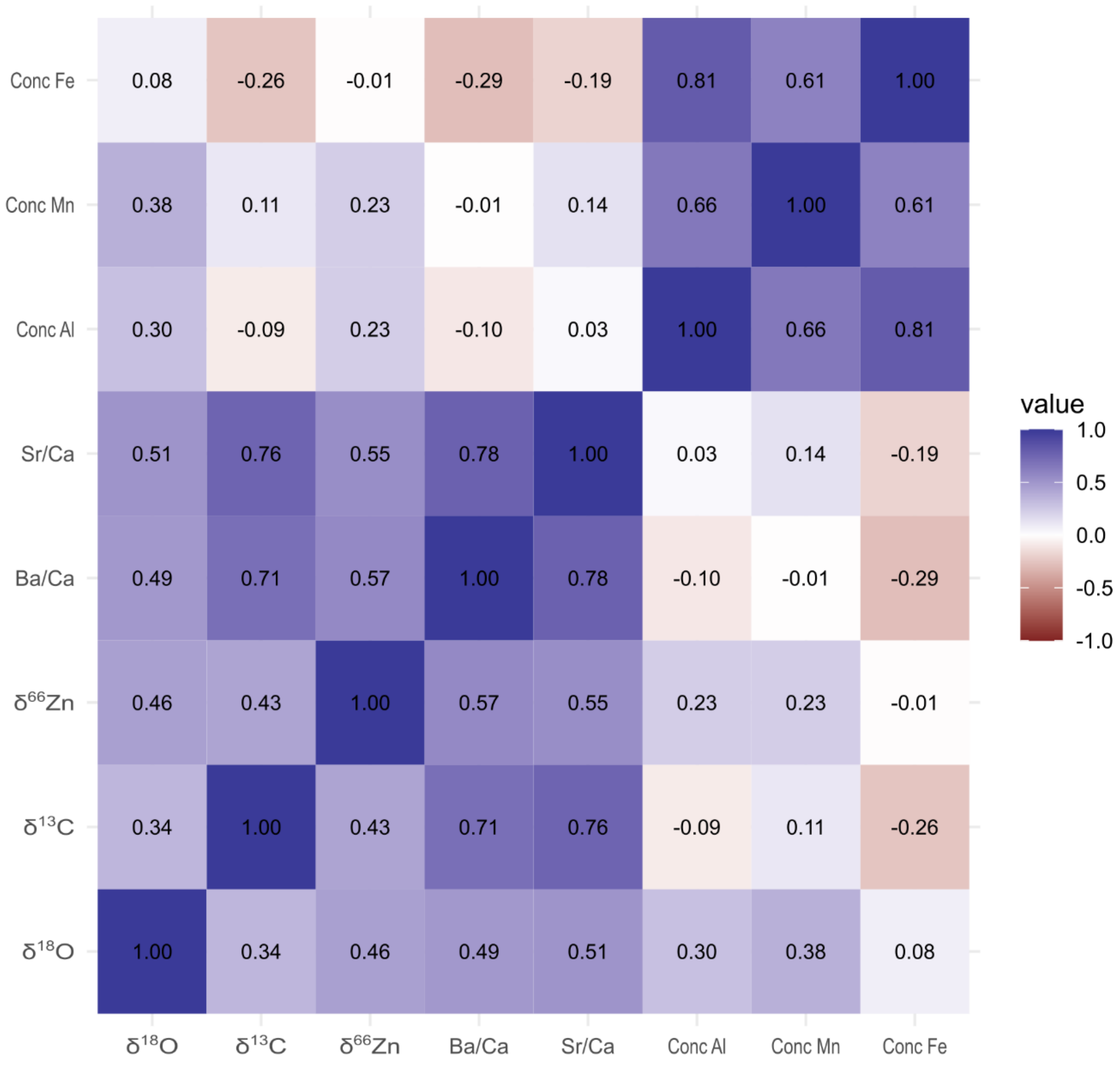

Figure S3. Spearman correlation heatmap of the isotopic and elemental values of Abu Tbeirah.

3.3 Enamel Carbonate Content

Enamel carbonate content is highly sensitive to diagenesis, with non-diagenetic values typically below 10% (35).
Two faunal samples exceeded this threshold:

- A1 (fish) and A19 (*Equus*).

However, no elevated levels of Al, Fe, or Mn were detected in A19, suggesting that the high $\%CO_3$ may not reflect substantial diagenetic alteration.

3.4 Zinc content in contaminated shells

Zinc concentrations in uncontaminated freshwater shells are typically low (~2 ppm). Contaminated samples show elevated Zn levels (~5 ppm), but this still represents less than 2% of the average Zn concentration measured in human enamel (~103 ppm). Therefore, even if minor contamination occurred, its influence on bulk $\delta^{66}Zn$ values would be negligible.

ARCH ID	ID	Species	Sample type	notes	mg	Mn	Al	P	Ca	Fe	Zn	Sr	Ba	As	Note
1983	1	*Homo sapiens*	Enamel	Dentine + Dirt ?	19.05	70.91	84.77	166020.16	331399.24	90.60	128.09	386.48	2.74	1.02	Good
1984	2	*Homo sapiens*	Enamel		6.42	50.31	134.30	131611.41	303874.34	90.60	101.02	697.56	3.65	5.06	Good
1985	3	*Homo sapiens*	Enamel		5.83	80.76	141.51	149463.75	341186.67	112.04	139.11	557.90	3.89	3.44	Good
1986	4	*Homo sapiens*	Enamel		6.62	8.01	83.34	150420.26	327515.95	28.59	114.18	323.51	1.54	0.57	Good
1987	5	*Homo sapiens*	Enamel		5.78	362.42	994.41	134004.86	299943.77	1191.59	65.32	238.99	13.00	1.36	Mn Al Fe Rich
1988	6	*Homo sapiens*	Enamel	small Dentine ?	5.09	77.11	104.50	144482.22	323849.08	76.31	108.78	376.51	3.69	1.79	Good
1989	7	*Homo sapiens*	Enamel	some Dirt	7.65	72.26	121.11	141330.70	304402.30	120.34	100.68	281.02	2.60	1.32	Good
1990	8	*Homo sapiens*	Enamel		6.36	38.28	81.42	157555.83	348660.97	45.09	55.59	391.59	3.59	1.51	Good
1991	9	*Homo sapiens*	Enamel	small Dentine ?	11.88	20.94	51.69	153158.65	293945.81	56.22	82.78	354.89	3.81	1.71	Good
1992	10	*Homo sapiens*	Enamel		5.68	442.47	91.84	165201.33	338448.37	133.68	196.11	449.56	11.83	3.87	Mn Rich
1993	11	*Homo sapiens*	Enamel		8.12	39.31	64.44	149489.25	310711.33	22.51	121.82	307.82	1.97	0.62	Good
1994	12	*Homo sapiens*	Enamel		6.44	86.44	83.55	157213.88	339976.83	28.73	136.66	466.20	4.49	1.34	Good
1995	13	*Homo sapiens*	Enamel		7.61	6.33	79.02	166539.19	334473.77	43.79	128.60	462.80	11.88	1.89	Good

1996	14	*Homo sapiens*	Enamel		15.98	4.84	34.16	147972.18	283620.40	22.18	64.20	421.71	2.35	1.80	Good
1997	15	*Homo sapiens*	Enamel		9.2	63.15	81.26	153380.51	311519.49	47.55	89.51	394.23	3.28	1.69	Good
1998	16	*Homo sapiens*	Enamel		10.26	5.97	52.70	155323.52	292986.09	23.82	93.90	267.95	2.32	1.97	Good
1999	17	*Homo sapiens*	Enamel	small Dentine ?	13.75	6.10	42.63	168228.68	298932.33	26.41	72.69	440.32	3.30	1.34	Good
2000	18	*Homo sapiens*	Enamel	Dirt	10.73	74.50	49.26	183276.34	324144.45	23.37	121.49	384.63	1.77	0.55	Good
2001	19	*Homo sapiens*	Enamel		13.85	18.79	40.06	183379.70	346698.37	22.08	84.72	397.48	1.25	0.63	Good
2002	20	*Homo sapiens*	Enamel		8.62	61.47	59.32	89249.22	182600.50	24.47	45.13	207.71	2.38	1.13	Good
2003	21	*Homo sapiens*	Enamel		6.74	30.95	269.45	153004.20	299127.68	186.07	48.48	266.83	3.61	0.62	Al Fe Rich
2004	22	*Homo sapiens*	Enamel		14.31	0.50	39.53	141295.92	267446.43	21.16	78.15	304.13	2.53	1.05	Good
2005	23	*Homo sapiens*	Enamel		7.74	677.25	90.23	192018.99	334773.29	201.47	146.45	439.91	3.95	2.31	Mn Fe Rich
2006	24	*Homo sapiens*	Enamel	Dirt	10.14	271.90	57.80	161790.96	277123.90	70.48	83.12	309.85	7.30	2.27	Mn Rich
2007	25	*Homo sapiens*	Enamel		6.27	70.22	117.50	184600.20	309290.12	72.43	165.78	235.58	3.69	0.46	Good
2008	26	*Homo sapiens*	Enamel		7.09	11.65	154.95	165895.52	304370.04	147.18	122.35	248.26	2.09	0.34	Al Rich
2009	28	*Homo sapiens*	Enamel		7.64	68.58	69.57	169681.73	315067.13	24.37	247.34	340.86	2.62	0.69	Good
2010	29	*Homo sapiens*	Enamel		8.6	33.09	73.26	153912.34	255514.72	38.63	46.41	462.62	3.88	2.85	Good

2011	30	*Homo sapiens*	Enamel		9.49	5.08	55.16	127922.87	229058.16	20.10	55.62	269.65	2.66	0.92	Good
2012	31	*Homo sapiens*	Enamel		6.61	110.93	79.03	154334.44	258632.69	19.08	66.23	283.46	2.24	0.72	Good
2013	32	*Homo sapiens*	Enamel		11.72	42.04	48.65	144689.63	246892.91	23.49	86.58	223.55	1.77	0.88	Good
2014	A1	Pisces	spine		27.65	6565.07	218.54	142035.97	284162.66	185.59	48.45	2046.07	113.98	29.69	Mn Al Fe Rich
2015	A2	*Bos taurus*	Enamel		9.19	55.71	64.72	142044.70	265606.06	26.84	26.85	810.50	15.97	2.14	Good
2016	A3	*Sus domesticus*	Enamel	small Dentine ?	15.55	17.43	49.66	114387.36	195018.39	37.39	19.48	538.45	7.07	5.02	Good
2017	A4	*Unio tigridis*	shell		17.07	55.86	122.23	-7220.76	269083.51	127.25	1.67	928.86	15.78	0.12	Good
2018	A6	*Unio tigridis*	shell		17.17	86.55	129.29	-6949.24	316086.63	143.82	3.47	1053.84	25.20	0.50	Good
2019	A7	*Unio tigridis*	shell	Yellow	17.18	29.67	69.15	-7591.15	331412.76	66.78	1.24	1055.66	24.36	0.13	Good
2020	A8	*Sus domesticus*	Enamel	yellow	34.68	2.50	23.29	149832.49	251764.42	24.97	28.02	552.39	4.55	1.44	Good
2021	A10	*Ovis* vel *Capra*	Enamel	Yellow	22.25	1.42	24.87	141265.43	248218.98	12.97	24.99	893.37	44.79	1.14	Good
2022	A11	Gastropoda	shell	Yellow	8.84	8.16	255.02	-14307.76	268021.94	224.16	3.35	2419.92	137.64	0.37	Al Fe Rich
2023	A12	*Bos taurus*	Enamel		46.63	1.99	13.03	153987.69	280177.74	16.77	30.33	728.66	26.93	1.34	Good
2024	A13	*Ovis* vel *Capra*	Enamel		13.34	5.01	71.10	152239.00	263226.21	53.84	34.27	1035.57	30.27	2.58	Good
2025	A15	*Ovis* vel *Capra*	Enamel		11.38	0.12	48.75	171080.95	293613.83	17.45	26.73	1283.78	33.82	2.86	Good

2026	A16	Gastropoda	shell		5.45	44.86	1087.89	-23137.23	284642.65	1239.76	11.52	1747.61	6.73	0.68	Al Fe Rich
2027	A17	*Ovis* vel *Capra*	Enamel		14.36	2.21	37.13	195821.20	325271.46	19.72	32.55	1248.32	44.70	2.00	Good
2028	A18	cf. *Ovis aries*	Enamel		11.06	252.44	125.92	193871.15	330839.58	179.78	60.40	1035.70	12.14	5.42	Mn Fe Rich
2029	A19	cf. *Equus* sp.	Enamel		20.28	0.49	28.81	318573.65	531741.30	30.58	33.81	2477.06	20.69	1.79	Good
2030	A20	cf. *Sus scrofa*	Enamel		6.22	424.86	227.94	216938.25	360574.50	347.26	41.80	947.54	12.57	4.83	Al Mn Fe Rich
2031	A22	*Sus domesticus*	Enamel	small Dentine ?	17.26	17.73	68.85	219968.24	381342.67	133.37	61.94	1380.69	9.53	38.79	Good
2033	A26	*Sus domesticus*	Enamel		11.42	29.11	52.08	347857.89	560418.11	39.23	59.92	1085.33	7.12	4.05	Good
2032	A24	*Ovis* vel *Capra*	Enamel		16.91	0.93	32.16	143154.63	229091.14	15.21	43.81	471.97	2.29	1.53	Good
2034	A27	*Equus asinus*	Enamel	small Dentine ?	36.96	10.90	48.56	151007.49	313397.47	135.70	90.58	3152.16	26.20	26.10	Good
2035	A28	*Ovis* vel *Capra*	Enamel		10.23	94.65	128.19	212108.69	363875.40	131.40	37.53	1709.62	55.97	6.45	Good
2036	A29	*Ovis* vel *Capra*	Enamel		11.51	270.57	185.39	200679.18	350472.28	192.35	34.96	1402.03	34.84	5.50	Al Mn Fe Rich
2037	A31	*Sus domesticus*	Enamel	some Dirt	13.63	1226.77	68.08	223227.92	360849.44	170.41	45.83	792.08	17.96	5.07	Mn Fe Rich

Table S2. Trace element concentrations in ppm (Zn, Sr, Ba) and potential contamination indicators (Fe, Mn, Al) for all samples, red values are over the non contamination limit.

Supplementary Text 4
Zinc, carbon and oxygen stable isotopes and Ba/Ca and Sr/Ca elemental ratios

4.1 Introduction to the proxy

Stable zinc isotope values ($\delta^{66}Zn$) measured on bioapatite can be used to infer dietary habits. This is because $\delta^{66}Zn$ values will deplete at every step of the food chain, with a decrease of around 0.3-0.4‰ (see, 36–39). As already suggested by paleodiet studies (40) and confirmed in recent experimental feeding studies (37), the mixing of all resources eaten dictates the isotopic values in consumers because of the absence of any marked isotopic fractionation upon Zn bioassimilation. Generally speaking, muscles and many soft tissues exhibit lower values compared to the average value of the body (notably, bones) and the diet (37, 40). In this way, it will be possible to differentiate between trophic position but also between bone-eating carnivores and meat-eaters (38). In plants, there is also a differentiation driven by fractionation, between higher-growing species and lower-growing ones (such as grasses), where the latter exhibit higher values (41). For this reason, in some cases, a differentiation between browsers and grazers emerged (39, 41). Generally, values associated with breastfeeding are enriched compared to post-weaning (42). Zn isotope ratios can be impacted by local geology and environmental context (38, 41, 43, 44); however, only to a small degree and not when the bedrock has a similar lithology (41).
As for stable carbon isotope values, it should be noted that $\delta^{13}C$ values vary depending on the photosynthetic pathways of plants (i.e., C_3, C_4 and CAM) as well as between terrestrial and marine environments (45). In mammals, the $\delta^{13}C$ values of enamel structural carbonate reflect the entire diet (all macronutrients), namely proteins, carbohydrates and lipids (46). This is because it precipitates in equilibrium with blood bicarbonate (46, 47). Therefore, $\delta^{13}C$ values from enamel and from bone collagen are not directly interchangeable (48). Enamel apatite's values can vary between 10‰ and 14‰ depending on physiological factors such as body mass and metabolic rate (47, 49). In general, human enamel values are enriched by approximately 12‰ compared to diet (49, 50). $\delta^{13}C$ values in plants are also influenced by environmental and climatic conditions. Notably, C_3 plants show a broader isotopic range and are more sensitive to environmental variability than C_4 plants (51). The isotopic response of C_3 plants is better understood and tends to increase with factors such as light intensity, nutrient availability, salinity, and water stress (48, 52–54). In particular, reduced mean annual precipitation is associated with increased $\delta^{13}C$ values (55). Lastly, breastfeeding can cause a $\delta^{13}C$ enrichment of approximately +1‰ in infants (56).
Stable oxygen isotope values ($\delta^{18}O$) measured in bioapatite are commonly used as proxies for geographic origin (57). This is because oxygen isotope values, especially for meteoric water, are influenced by geographic and climatic variables such as latitude, altitude, distance from the coast, and temperature (48, 57–59). In general, $\delta^{18}O$ values decrease with increasing distance from the coast (60), elevation, and latitude (48, 61). Evaporative processes enrich $\delta^{18}O$ values by preferentially removing the lighter isotope (^{16}O), particularly in standing or shallow bodies of water (62). Wetland plants such as reeds often show elevated $\delta^{18}O$ due to evapotranspiration (13). The extent of evaporation-related enrichment also depends on the size and hydrology of the water body.
In living organisms, $\delta^{18}O$ values are influenced by drinking water, water vapor, and the water content in food (63). For humans and animals, the $\delta^{18}O$ signature of drinking water is largely determined by precipitation-fed sources like groundwater and surface water. River water, by contrast, reflects broader climatic inputs, including regional recharge and not just local rainfall (64). Among animals, obligate drinkers are more directly influenced by the $\delta^{18}O$ of drinking water (65), whereas non-obligate drinkers may show isotope signatures more strongly affected by food moisture content (66). In humans, by contrast, culturally mediated factors such as stewing, boiling, brewing, and distillation can alter $\delta^{18}O$ values significantly. Experimental studies suggest that boiling increases $\delta^{18}O$ by 0.3‰ brewing, 1.3‰ milk, 2.3‰ stewing, 3.7‰, and distillation by 6.5‰ to the source water (57). Breastfeeding can also result in $\delta^{18}O$ enrichment in infants, typically around +1.5‰ (56).
However, isolating a single causal factor is often challenging due to the interplay of multiple environmental and cultural influences (48).
Among the trace elements analyzed, strontium (Sr) and barium (Ba), both of which correlate with calcium (Ca), are particularly informative for reconstructing dietary habits (67, 68). Sr and Ba enter the body through diet and can substitute Ca due to their similar chemical properties. Their use in dietary studies relies on the principle of biopurification of calcium, whereby Ba and Sr are preferentially excreted during metabolic processes (68, 69). In contrast, Ca, being essential, is homeostatically regulated by the body and remains relatively stable regardless of dietary input.

Because plants generally contain higher concentrations of Sr and Ba, the ratios of Sr/Ca and Ba/Ca decrease progressively up the trophic chain. Consequently, these ratios can serve as trophic level indicators: herbivores and grazers typically show higher Sr/Ca and Ba/Ca values than carnivores and browsers (70). In addition to trophic level, Sr/Ca and Ba/Ca ratios are also sensitive to early-life feeding practices. In utero, both ratios tend to be low. During exclusive breastfeeding, Sr/Ca values typically decrease, while Ba/Ca values tend to increase due to differences in transfer efficiency and metabolic handling. During weaning, the introduction of solid, plant-based foods leads to a rise in both Sr/Ca and Ba/Ca. Finally, in the post-weaning phase, Sr/Ca tends to decrease if meat becomes a dominant dietary component, while Ba/Ca values also decrease (71).

4.2 Accuracy of Zn, C and O isotope measurements

We measured one external standard for Zn isotope (SRM 1400). Its values are indistinguishable from other measures in published studies (Table S3). All analytical duplicates from a single aliquot had $\delta^{66}Zn$ values with SD ≤ 0.05 ‰ (Table S4).

**this study**		**$\delta^{66}Zn$ (‰- JMC Lyon)**	**1SD**
	SRM 1400 MG1	0.95	0.04
	SRM 1400 MG2	0.91	0.05
	SRM 1400 MG3	0.90	0.02
	SRM 1400 (average) N=13	0.93	0.04
**other studies**			
	Jaouen et al., 2022	0.92	0.05
	Jaouen et al., 2018	1	0.04
	Bourgon et al., 2020	0.94	0.05
	Bourgon et al., 2021	0.96	0.01
	McCormack et al., 2021	0.95	0.03
	Jaouen et al., 2020	1	0.07
	Moubtahij et al., 2024	0.93	0.03

Table S3. Zinc isotope values ($\delta^{66}Zn$) for the reference materials used in this study, compared with published values from the literature (36, 38, 40, 42, 43, 72, 73)

ID	Species	Tooth	$\delta^{66}Zn$	1SD
1	*Homo sapiens*	ULM1	0.75	
1	*Homo sapiens*	ULM1	0.75	
	Mean		0.75	0.00
2	*Homo sapiens*	URM1	0.97	
3	*Homo sapiens*	LLC	0.73	
4	*Homo sapiens*	URI1	0.67	
5	*Homo sapiens*	URm2	0.80	
6	*Homo sapiens*	URm2	0.59	
6	*Homo sapiens*	URm2	0.57	
	Mean		0.58	0.01
7	*Homo sapiens*	i	0.71	
8	*Homo sapiens*	LLm2	0.58	
9	*Homo sapiens*	URi2	0.79	
9	*Homo sapiens*	URi2	0.81	
	Mean		0.80	0.02
10	*Homo sapiens*	ULP3	0.68	
11	*Homo sapiens*	LRC	0.63	
11	*Homo sapiens*	LRC	0.68	
	Mean		0.66	0.03
12	*Homo sapiens*	LRC	0.61	
12	*Homo sapiens*	LRC	0.61	
	Mean		0.61	0.00
13	*Homo sapiens*	URC	0.61	
13	*Homo sapiens*	URC	0.60	

	Mean		0.61	0.01
14	*Homo sapiens*	URP4	0.79	
14	*Homo sapiens*	URP4	0.81	
	Mean		0.80	0.02
15	*Homo sapiens*	LRc	0.68	
16	*Homo sapiens*	ULc	0.98	
16	*Homo sapiens*	ULc	0.99	
	Mean		0.98	0.01
17	*Homo sapiens*	LRP3	0.61	
17	*Homo sapiens*	LRP3	0.66	
	Mean		0.64	0.04
18	*Homo sapiens*	URC	0.62	
18	*Homo sapiens*	URC	0.70	
18	*Homo sapiens*	URC	0.72	
	Mean		0.68	0.05
20	*Homo sapiens*	LRP3	0.58	
21	*Homo sapiens*	Rm1	0.73	
22	*Homo sapiens*	LRM1	0.70	
23	*Homo sapiens*	URc	0.89	
24	*Homo sapiens*	LRP3	0.77	
25	*Homo sapiens*	RM1	0.71	
26	*Homo sapiens*	LM1	0.82	
26	*Homo sapiens*	LM1	0.83	
	Mean		0.82	0.01
27	*Homo sapiens*	LM3	0.61	

27	*Homo sapiens*	LM3	0.61	
	Mean		0.61	0.01
28	*Homo sapiens*	LM1	0.68	
28	*Homo sapiens*	LM1	0.74	
	Mean		0.71	0.04
29	*Homo sapiens*	i	0.63	
30	*Homo sapiens*	i1	0.66	
30	*Homo sapiens*	i1	0.61	
	Mean		0.64	0.03
31	*Homo sapiens*	UP	0.68	
32	*Homo sapiens*	LC	0.66	
32	*Homo sapiens*	LC	0.72	
	Mean		0.69	0.04
A1	Pisces	spine	0.66	
A2	*Bos taurus*	UM	1.04	
A3	*Sus domesticus*	LP3	0.78	
A8	*Sus domesticus*	M3	0.78	
A8	*Sus domesticus*	M3	0.82	
	Mean		0.80	0.02
A10	*Ovis* vel *Capra*	I3	1.26	
A10	*Ovis* vel *Capra*	I3	1.24	
	Mean		1.25	0.02
A12	*Bos taurus*	UP3-4	1.08	
A13	*Ovis* vel *Capra*		1.31	
A15	*Ovis* vel *Capra*	M2	1.27	

A17	*Ovis* vel *Capra*	P4	1.26	
A18	cf. *Ovis aries*	LD4	0.94	
A19	cf. *Equus* sp.	tooth	0.90	
A20	cf. *Sus scrofa*	UI	0.73	
A22	*Sus domesticus*	LP2	0.84	
A22	*Sus domesticus*	LP2	0.77	
	Mean		0.81	0.05
A25	*Ovis* vel *Capra*	UM	0.84	
A25	*Ovis* vel *Capra*	UM	0.81	
	Mean		0.83	0.02
A27	*Equus asinus*	LM3	1.58	
A28	*Ovis* vel *Capra*		1.27	
A29	*Ovis* vel *Capra*	UM2	1.20	
A31	*Sus domesticus*	Up4	0.91	

Table S4. Duplicate and triplicate measurements of zinc isotope ratios ($\delta^{66}Zn$) for selected samples to assess analytical reproducibility

Four reference materials were run along with samples during C and O isotope analyses (IA-R066, NBS 18, Y-02, IA-R022), which gave the following quality parameters:

**Check Sample**		**$\delta^{13}C_{V-PDB}$ (‰)**	**$\delta^{18}O_{V-PDB}$ (‰)**
NBS18	Mean	-5.01	-23.20
Carbonatite	St. Dev.	0.01	0.07
	Accepted Value	-5.01	-23.20
IA-R066	Mean	2.33	-1.52
Chalk	St. Dev.	0.06	0.01

	Accepted Value	2.33	-1.52
Y-02	Mean	1.36	-9.66
Calcite	St. Dev.	0.00	0.14
	Long Term Mean Value	1.48	-9.59
IA-R022	Mean	-28.67	-22.68
Calcium Carbonate	St. Dev.	0.14	0.18
	Accepted Value	-28.63	-22.69

Table S5. Reference materials used for carbon and oxygen isotope analyses, including standards for calibration and quality control

4.3 Accuracy of trace elements measurement

For the trace elements, the multi-element standard solution EPOND and the reference material SLRS6 (74) were measured along with the samples. Ca gave higher concentrations for EPOND and fair for SLRS6. The other elements gave concentrations coherent with the expected range.

	Mn	Al	Ca	Fe	Zn	Sr	Ba
SLRS6-N	1.97	35.67	7963.00	79.22	1.51	39.42	14.62
Expected	2.12	33.80	8760.00	84.30	1.76	40.66	14.28
SD	0.06	2.20	200.00	3.60	0.12	0.32	0.48
EPOND-BT	951.37	1025.67	3945.47	940.82	817.28	955.34	1013.28
expected	1000	1000	1000	1000	1000	1000	1000
SD	5	5	5	5	5	5	5

Table S6. Trace element concentrations (in ppb) for the reference material SLRS6 and the standard solution EPOND used in elemental analysis.

4.4 Sites with available post-weaning zinc isotope

The availability of published zinc isotope data is notably limited and becomes even more so when considering only post-weaning teeth. The resulting dataset encompasses sites that differ substantially in chronology, geography, and sociocultural context.

Taforalt (36): Situated in an arid North African environment. The Taforalt assemblage corresponds to the Epipaleolithic period. Archaeological evidence indicates no discernible differentiation in social status among individuals. The environmental and cultural context suggests subsistence strategies adapted to harsh, resource-limited conditions.

Rennes (43): Located in a temperate European setting. Rennes assemblage dates to the medieval period and is associated with a socially privileged population. Material culture and historical records indicate higher socioeconomic status, reflected in access to resources and living conditions, consistent with the broader societal hierarchies of the time.
Saint Laurent (75): This site is in a temperate mountainous region and represents a contemporary urban neighborhood of popular character. The social composition is heterogeneous, reflecting modern demographic patterns and the dynamics of a densely inhabited residential area. Environmental factors and urban development have shaped both the lifestyle and the built environment of the community.
Lapa do Santo (42): This site is in a tropical region and presents early to middle Pleistocene occupations. The inhabitants were associated with the gathering and hunting of small and medium-sized fauna.

ID	species	$\delta^{13}C$	$\delta^{18}O$	Ba/Ca	Sr/Ca	$\delta^{66}Zn$
1	*Homo sapiens*	-11.32	-5.17	-5.08329	-2.93322	0.75
2	*Homo sapiens*	-12.58	-4.44	-4.92007	-2.63911	0.97
3	*Homo sapiens*	-11.56	-3.46	-4.94264	-2.78643	0.73
4	*Homo sapiens*	-10.83	-4.3	-5.32899	-3.00535	0.67
5	*Homo sapiens*	NA	NA	-4.36311	-3.09866	0.8
6	*Homo sapiens*	-11.63	-3.44	-4.94298	-2.93457	0.58
7	*Homo sapiens*	-8.79	-4.78	-5.06931	-3.03471	0.71
8	*Homo sapiens*	-11.95	-3.14	-4.98769	-2.94957	0.58
9	*Homo sapiens*	-11.62	-3.09	-4.88752	-2.91817	0.8
10	*Homo sapiens*	NA	NA	-4.45652	-2.87671	0.68
11	*Homo sapiens*	NA	NA	-5.19894	-3.00406	0.66
12	*Homo sapiens*	-10.81	-4.53	-4.87889	-2.86288	0.61
13	*Homo sapiens*	-11.21	0.57	-4.44958	-2.85897	0.61
14	*Homo sapiens*	-11.9	-0.24	-5.08146	-2.82772	0.8
15	*Homo sapiens*	-10.33	-4.42	-4.97721	-2.89774	0.68
16	*Homo sapiens*	-12.23	0.06	-5.102	-3.03879	0.98
17	*Homo sapiens*	-11.56	-4.27	-4.95764	-2.83181	0.64
18	*Homo sapiens*	-11.41	-4.58	-5.26286	-2.9257	0.68
19	*Homo sapiens*	-11.45	-4.85	-5.44307	-2.94063	0.58

20	*Homo sapiens*	-11.97	-1.63	-4.88486	-2.94404	0.73
21	*Homo sapiens*	-11.19	-5.87	-4.91837	-3.04962	0.7
22	*Homo sapiens*	-11.55	-1.03	-5.0246	-2.94418	0.89
23	*Homo sapiens*	-11.49	-4.82	-4.92835	-2.88139	0.77
24	*Homo sapiens*	-12.22	-1.53	-4.57916	-2.95152	0.71
25	*Homo sapiens*	-11.5	-4.18	-4.92278	-3.11823	0.82
26	*Homo sapiens*	-11.87	-5.37	-5.16338	-3.0885	0.61
27	*Homo sapiens*	-10.72	-3.96	NA	NA	NA
28	*Homo sapiens*	-12.28	-4.84	-5.08076	-2.96583	0.71
29	*Homo sapiens*	-11.21	-3.61	-4.81866	-2.74219	0.63
30	*Homo sapiens*	-11.6	-2.9	-4.9346	-2.92914	0.64
31	*Homo sapiens*	-11.67	-4.42	-5.06288	-2.96019	0.68
32	*Homo sapiens*	-11.76	-4.13	-5.14412	-3.04313	0.69
A1	Pisces	-3.26	0.46	-3.39673	-2.14265	0.66
A2	*Bos taurus*	-2.32	-1.74	-4.22107	-2.51548	1.04
A3	*Sus domesticus*	-8.62	-0.77	-4.44079	-2.55893	0.78
A4	*Unio tigridis*	-5.85	-1.94	-4.23186	-2.46194	
A6	*Unio tigridis*	-5.95	-6.78	-4.09841	-2.47703	
A7	*Unio tigridis*	-3.35	-6.58	-4.13367	-2.49685	
A8	*Sus domesticus*	-9.74	0.93	-4.74279	-2.65875	0.8
A10	*Ovis* vel *Capra*	-4.18	-0.16	-3.74361	-2.4438	1.25
A11	Gastropoda	-2.58	1.94	-3.28944	-2.04437	
A12	*Bos taurus*	-3.38	-0.72	-4.01721	-2.58491	1.08
A13	*Ovis* vel *Capra*	-5.56	-1.87	-3.93933	-2.40515	1.31
A15	*Ovis* vel *Capra*	NA	NA	-3.93863	-2.35928	1.27

A16	Gastropoda	2.45	0.54	-4.62599	-2.21186	
A17	*Ovis* vel *Capra*	-5.87	-0.33	-3.86196	-2.41592	1.26
A18	cf. *Ovis aries*	-5.26	2.78	-4.43549	-2.50439	0.94
A19	cf. *Equus* sp.	-6.94	1.62	-4.40996	-2.33176	0.9
A20	cf. *Sus scrofa*	NA	NA	-4.45762	-2.5804	0.73
A22	*Sus domesticus*	-8.1	1.47	-4.60237	-2.44122	0.81
A24	*Sus domesticus*	-9.31	-3.95	-5.00041	-2.68609	0.83
A26	*Sus domesticus*	-10.67	0.83	-4.89609	-2.71295	
A27	*Equus asinus*	-4.8	-1.1	-4.07786	-1.99749	1.58
A28	*Ovis* vel *Capra*	-6.38	-0.81	-3.81302	-2.32805	1.27
A29	*Ovis* vel *Capra*	-7.85	-3.92	-4.0026	-2.3979	1.2
A31	*Sus domesticus*	-11.57	-0.82	-4.30311	-2.65856	0.91

Table S7. Isotopic values ($\delta^{66}Zn$, $\delta^{13}C$, $\delta^{18}O$) and elemental concentration ratios (Sr/Ca, Ba/Ca) for all human and faunal samples from Abu Tbeirah.

Supplementary Text 5: Statistical tests and investigations

All statistical analyses and plotting were conducted in R version 4.1.2 (76), following the guidelines proposed by Vaiglova et al. (2023) (77). Data visualization was conducted using the ggplot2 package (78), and descriptive statistics were generated with dplyr (79) and Hmisc (80).

The complete code used to perform the analysis and plot the data is available in the Zenodo database (10.5281/zenodo.17868175) to comply with the FAIR principles.

Boxplots were used to identify potential outliers, defined as values outside 1.5 × the interquartile range (IQR, Figure S10).

To account for unequal variances and unbalanced sample sizes across groups, Welch's ANOVA was employed, a heteroscedasticity-robust alternative to standard one-way ANOVA. Welch's method relaxes the assumption of homoscedasticity and maintains accurate Type I error rates even when group sizes are small or markedly unequal (81). For post-hoc pairwise comparisons, we used the Games–Howell procedure, which does not require equal variances or equal sample sizes (82).

The significance threshold (α) was set at 0.05.

Following Vaiglova et al. (2023) (77) and Giaccari et al. (2024) (83), statistical tests were complemented with 95% confidence intervals (CIs) for group means (84), following the approach proposed by Cumming 2009 (85), suggesting that non-overlapping 95% CIs for independent samples approximate p-values ≤ 0.01. Additionally, CI width was used as a qualitative proxy for statistical power, that consists in the probability of correctly rejecting a false null hypothesis (H_0) (86).

Statistical tests and CIs are presented in the Table S8 with all the individuals and Table S9 without statistical outliers.

Isotope	Category	Group 1	Group 2	n 1	n 2	G1 CIs Lower	G1 CIs Upper	G2 CIs Lower	G2 CIs Upper	Estimate	Statistic	df	P.adj	Effect Size
$\delta^{66}Zn$	Species	*Homo sapiens*	*Ovis* vel *Capra*	31	6	0.68	0.75	1.23	1.29	0.5	23.2	23.8	<0.001	-5.7
$\delta^{66}Zn$	Species	*Homo sapiens*	*Sus domesticus*	31	5	0.68	0.75	0.79	0.87	0.1	3.9	10.6	0.007	-1.2
$\delta^{66}Zn$	Species	*Ovis* vel *Capra*	*Sus domesticus*	6	5	1.23	1.29	0.79	0.87	-0.4	16.2	7.1	<0.001	10.1
$\delta^{66}Zn$	Age	Adult	Non-Adult	16	15	0.65	0.74	0.68	0.79	0.0	1.0	27.7	0.578	-0.4
$\delta^{66}Zn$	Building	Animal	Building A1	18	5	0.92	1.16	0.63	0.77	-0.3	4.6	19.6	0.001	1.4
$\delta^{66}Zn$	Building	Animal	Building A2	18	12	0.92	1.16	0.63	0.71	-0.4	5.7	20.9	<0.001	1.8
$\delta^{66}Zn$	Building	Animal	Cemetery Area 1	18	8	0.92	1.16	0.67	0.74	-0.3	5.3	19.6	<0.001	1.5
$\delta^{66}Zn$	Building	Building A1	Building A2	5	12	0.63	0.77	0.63	0.71	0.0	0.8	6.5	0.865	0.4
$\delta^{66}Zn$	Building	Building A1	Cemetery Area 1	5	8	0.63	0.77	0.67	0.74	0.0	0.0	5.6	1.000	0.0
$\delta^{66}Zn$	Building	Building A2	Cemetery Area 1	12	8	0.63	0.71	0.67	0.74	0.0	1.3	18.0	0.582	-0.5
$\delta^{66}Zn$	Alimentation	Mother	Probable Exclusive Breastfeeding	7	5	0.68	0.85	0.62	0.91	0.0	0.0	6.6	1.000	0.0
$\delta^{66}Zn$	Alimentation	Mother	Post lactation	7	11	0.68	0.85	0.90	1.20	0.3	3.0	14.7	0.060	-1.2
$\delta^{66}Zn$	Alimentation	Mother	Post weaning	7	14	0.68	0.85	0.64	0.70	-0.1	1.9	7.6	0.395	1.1
$\delta^{66}Zn$	Alimentation	Mother	Probable Post weaning	7	4	0.68	0.85	0.71	0.92	0.0	0.2	6.2	0.999	-0.1
$\delta^{66}Zn$	Alimentation	Probable Exclusive Breastfeeding	Post lactation	5	11	0.62	0.91	0.90	1.20	0.3	2.5	11.8	0.166	-1.1
$\delta^{66}Zn$	Alimentation	Probable Exclusive Breastfeeding	Post weaning	5	14	0.62	0.91	0.64	0.70	-0.1	1.1	4.3	0.786	0.9
$\delta^{66}Zn$	Alimentation	Probable Exclusive Breastfeeding	Probable Post weaning	5	4	0.62	0.91	0.71	0.92	0.0	0.2	6.9	1.000	-0.1

$\delta^{66}Zn$	Alimentation	Post lactation	Post weaning	11	14	0.90	1.20	0.64	0.70	-0.4	4.4	10.8	0.007	2.0
$\delta^{66}Zn$	Alimentation	Post lactation	Probable Post weaning	11	4	0.90	1.20	0.71	0.92	-0.3	2.6	11.9	0.139	1.1
$\delta^{66}Zn$	Alimentation	Post weaning	Probable Post weaning	14	4	0.64	0.70	0.71	0.92	0.1	1.7	3.4	0.537	-1.4
$\delta^{66}Zn$	Sex	F	M	6	7	0.63	0.81	0.63	0.74	0.0	0.3	7.9	0.994	0.1
$\delta^{66}Zn$	Sex	F	ND	6	18	0.63	0.81	0.68	0.78	0.0	0.4	7.0	0.974	-0.2
$\delta^{66}Zn$	Sex	M	ND	7	18	0.63	0.74	0.68	0.78	0.0	1.0	13.9	0.726	-0.4
$\delta^{66}Zn$	Grave Goods	No	pottery vessels	4	9	0.70	0.80	0.67	0.80	0.0	0.6	9.6	0.968	0.3
$\delta^{66}Zn$	Grave Goods	No	pottery vessels, Reed basket	4	3	0.70	0.80	0.61	0.73	-0.1	1.7	4.7	0.488	1.3
$\delta^{66}Zn$	Grave Goods	No	unknown	4	9	0.70	0.80	0.62	0.71	-0.1	2.4	6.6	0.235	1.3
$\delta^{66}Zn$	Grave Goods	pottery vessels	pottery vessels, Reed basket	9	3	0.67	0.80	0.61	0.73	-0.1	1.0	6.9	0.826	0.5
$\delta^{66}Zn$	Grave Goods	pottery vessels	unknown	9	9	0.67	0.80	0.62	0.71	-0.1	1.5	14.1	0.571	0.7
$\delta^{66}Zn$	Grave Goods	pottery vessels, Reed basket	unknown	3	9	0.61	0.73	0.62	0.71	0.0	0.3	4.3	0.996	0.2
$\delta^{66}Zn$	Feeding Behaviour	Grazer	Human	5	31	0.94	1.35	0.68	0.75	-0.4	3.2	4.2	0.100	2.9
$\delta^{66}Zn$	Feeding Behaviour	Grazer	Omnivore	5	6	0.94	1.35	0.77	0.86	-0.3	2.4	4.3	0.213	1.6
$\delta^{66}Zn$	Feeding Behaviour	Grazer	Ovicaprines	5	6	0.94	1.35	1.23	1.29	0.2	1.2	4.1	0.641	-0.8
$\delta^{66}Zn$	Feeding Behaviour	Human	Omnivore	31	6	0.68	0.75	0.77	0.86	0.1	3.2	11.8	0.035	-1.0
$\delta^{66}Zn$	Feeding Behaviour	Human	Ovicaprines	31	6	0.68	0.75	1.23	1.29	0.5	23.2	23.8	<0.001	-5.7
$\delta^{66}Zn$	Feeding Behaviour	Omnivore	Ovicaprines	6	6	0.77	0.86	1.23	1.29	0.5	15.8	8.2	<0.001	-9.1
$\delta^{66}Zn$	Drinking Strategy	Human	Non-Obligate Drinker	31	7	0.68	0.75	1.12	1.28	0.5	9.9	7.9	<0.001	-4.7

$\delta^{66}Zn$	Drinking Strategy	Human	Obligate Drinker	31	10	0.68	0.75	0.82	1.11	0.2	2.9	10.0	0.040	-1.6
$\delta^{66}Zn$	Drinking Strategy	Non-Obligate Drinker	Obligate Drinker	7	10	1.12	1.28	0.82	1.11	-0.3	2.9	13.9	0.029	1.3
$\delta^{13}C$	Species	*Homo sapiens*	Ovis vel Capra	29	6	-11.68	-11.16	-6.92	-5.19	5.4	10.5	5.7	<0.001	-6.7
$\delta^{13}C$	Species	*Homo sapiens*	Sus domesticus	29	6	-11.68	-11.16	-10.67	-8.79	1.8	3.3	5.6	0.089	-2.2
$\delta^{13}C$	Species	*Homo sapiens*	Unio tigridis	29	4	-11.68	-11.16	-7.52	-4.00	5.7	5.8	3.1	0.036	-6.2
$\delta^{13}C$	Species	*Ovis vel Capra*	*Sus domesticus*	6	6	-6.92	-5.19	-10.67	-8.79	-3.6	5.0	10.0	0.004	2.9
$\delta^{13}C$	Species	*Ovis vel Capra*	*Unio tigridis*	6	4	-6.92	-5.19	-7.52	-4.00	0.3	0.2	4.6	0.999	-0.2
$\delta^{13}C$	Species	*Sus domesticus*	*Unio tigridis*	6	4	-10.67	-8.79	-7.52	-4.00	3.9	3.5	4.8	0.085	-2.5
$\delta^{13}C$	Age	Adult	Non-Adult	16	15	-11.90	-11.31	-11.70	-10.88	0.3	0.9	25.1	0.880	-0.3
$\delta^{13}C$	Building	Building A1	Building A2	5	11	-11.77	-9.92	-11.56	-10.97	-0.2	0.3	4.7	0.991	0.2
$\delta^{13}C$	Building	Building A1	Cemetery Area 1	5	7	-11.77	-9.92	-11.94	-11.45	-0.6	1.0	4.5	0.760	0.7
$\delta^{13}C$	Building	Building A2	Cemetery Area 1	11	7	-11.56	-10.97	-11.94	-11.45	-0.4	2.0	15.9	0.243	0.9
$\delta^{13}C$	Alimentation	Mother	Probable Exclusive Breastfeeding	7	4	-11.65	-8.58	-12.09	-11.59	-1.6	1.6	6.3	0.718	0.8
$\delta^{13}C$	Alimentation	Mother	Post lactation	7	11	-11.65	-8.58	-8.44	-5.76	3.1	2.6	12.7	0.230	-1.3
$\delta^{13}C$	Alimentation	Mother	Post weaning	7	12	-11.65	-8.58	-11.65	-11.25	-1.2	1.3	6.1	0.889	0.8
$\delta^{13}C$	Alimentation	Mother	Weaning	7	3	-11.65	-8.58	-11.98	-10.72	-1.2	1.2	7.8	0.923	0.6
$\delta^{13}C$	Alimentation	Mother	Probable Post weaning	7	4	-11.65	-8.58	-12.43	-11.26	-1.6	1.6	7.5	0.758	0.8
$\delta^{13}C$	Alimentation	Probable Exclusive Breastfeeding	Post lactation	4	11	-12.09	-11.59	-8.44	-5.76	4.7	6.3	10.9	0.001	-2.2
$\delta^{13}C$	Alimentation	Probable Exclusive	Post weaning	4	12	-12.09	-11.59	-11.65	-11.25	0.4	2.0	5.9	0.530	-1.1

		Breastfeeding												
δ^{13}C	Alimentation	Probable Exclusive Breastfeeding	Weaning	4	3	-12.09	-11.59	-11.98	-10.72	0.4	0.8	2.5	0.980	-0.7
δ^{13}C	Alimentation	Probable Exclusive Breastfeeding	Probable Post weaning	4	4	-12.09	-11.59	-12.43	-11.26	0.0	0.0	4.2	1.000	0.0
δ^{13}C	Alimentation	Post lactation	Post weaning	11	12	-8.44	-5.76	-11.65	-11.25	-4.3	5.8	10.4	0.002	2.5
δ^{13}C	Alimentation	Post lactation	Weaning	11	3	-8.44	-5.76	-11.98	-10.72	-4.3	5.1	11.2	0.005	1.9
δ^{13}C	Alimentation	Post lactation	Probable Post weaning	11	4	-8.44	-5.76	-12.43	-11.26	-4.7	5.8	12.8	0.001	2.2
δ^{13}C	Alimentation	Post weaning	Weaning	12	3	-11.65	-11.25	-11.98	-10.72	0.0	0.0	2.2	1.000	0.0
δ^{13}C	Alimentation	Post weaning	Probable Post weaning	12	4	-11.65	-11.25	-12.43	-11.26	-0.4	1.1	3.5	0.935	0.8
δ^{13}C	Alimentation	Weaning	Probable Post weaning	3	4	-11.98	-10.72	-12.43	-11.26	-0.4	0.7	4.2	0.995	0.5
δ^{13}C	Sex	F	M	6	8	-12.05	-11.09	-12.15	-11.39	-0.2	0.6	10.4	0.934	0.3
δ^{13}C	Sex	F	ND	6	17	-12.05	-11.09	-11.65	-10.88	0.2	0.7	10.9	0.884	-0.3
δ^{13}C	Sex	M	ND	8	17	-12.15	-11.39	-11.65	-10.88	0.5	1.5	18.3	0.445	-0.6
δ^{13}C	Grave Goods	pottery vessels	pottery vessels, Reed basket	9	3	-12.03	-11.41	-11.97	-10.33	0.7	1.3	2.5	0.625	-1.1
δ^{13}C	Grave Goods	pottery vessels	unknown	9	9	-12.03	-11.41	-11.70	-11.19	0.3	1.2	15.4	0.628	-0.6
δ^{13}C	Grave Goods	pottery vessels, Reed basket	unknown	3	9	-11.97	-10.33	-11.70	-11.19	-0.4	0.8	2.3	0.849	0.8
δ^{13}C	Feeding Behaviour	Freshwater	Grazer	6	5	-6.42	-3.38			0.3	0.3	9.0	1.000	-0.2
δ^{13}C	Feeding Behaviour	Freshwater	Human	6	29	-6.42	-3.38	-11.68	-11.16	-6.6	7.5	5.2	0.004	6.3

$\delta^{13}C$	Feeding Behaviour	Freshwater	Omnivore	6	6	-6.42	-3.38	-10.67	-8.79	-4.8	4.8	8.2	0.011	2.7
$\delta^{13}C$	Feeding Behaviour	Freshwater	Ovicaprines	6	6	-6.42	-3.38	-6.92	-5.19	-1.2	1.2	8.0	0.815	0.7
$\delta^{13}C$	Feeding Behaviour	Grazer	Human	5	29	-5.84	-3.12	-11.68	-11.16	-6.9	8.6	4.2	0.005	7.6
$\delta^{13}C$	Feeding Behaviour	Grazer	Omnivore	5	6	-5.84	-3.12	-10.67	-8.79	-5.1	5.4	7.2	0.008	3.4
$\delta^{13}C$	Feeding Behaviour	Grazer	Ovicaprines	5	6	-5.84	-3.12	-6.92	-5.19	-1.5	1.6	6.9	0.608	1.0
$\delta^{13}C$	Feeding Behaviour	Human	Omnivore	29	6	-11.68	-11.16	-10.67	-8.79	1.8	3.3	5.6	0.112	-2.2
$\delta^{13}C$	Feeding Behaviour	Human	Ovicaprines	29	6	-11.68	-11.16	-6.92	-5.19	5.4	10.5	5.7	<0.001	-6.7
$\delta^{13}C$	Feeding Behaviour	Omnivore	Ovicaprines	6	6	-10.67	-8.79	-6.92	-5.19	3.6	5.0	10.0	0.005	-2.9
$\delta^{13}C$	Drinking Strategy	Freshwater	Non-Obligate Drinker	6	7	-6.42	-3.38	-6.74	-5.17	-1.1	1.1	7.4	0.782	0.7
$\delta^{13}C$	Drinking Strategy	Freshwater	Obligate Drinker	6	10	-6.42	-3.38	-9.36	-5.78	-2.7	2.1	13.6	0.292	1.0
$\delta^{13}C$	Drinking Strategy	Human	Non-Obligate Drinker	29	7	-11.68	-11.16	-6.74	-5.17	5.5	12.1	7.2	<0.001	-6.8
$\delta^{13}C$	Drinking Strategy	Human	Obligate Drinker	29	10	-11.68	-11.16	-9.36	-5.78	3.9	3.9	9.3	0.021	-2.4
$\delta^{13}C$	Drinking Strategy	Non-Obligate Drinker	Obligate Drinker	7	10	-6.74	-5.17	-9.36	-5.78	-1.6	1.5	12.1	0.594	0.6
$\delta^{18}O$	Species	*Homo sapiens*	Ovis vel Capra	29	6	-4.14	-2.86	-2.43	-0.26	2.3	3.4	7.9	0.054	-1.4
$\delta^{18}O$	Species	*Homo sapiens*	Sus domesticus	29	6	-4.14	-2.86	-2.08	0.89	3.1	3.6	6.6	0.051	-1.8
$\delta^{18}O$	Species	*Homo sapiens*	Unio tigridis	29	4	-4.14	-2.86	-6.68	-2.02	-0.8	0.6	3.3	0.968	0.5
$\delta^{18}O$	Species	*Ovis vel Capra*	*Sus domesticus*	6	6	-2.43	-0.26	-2.08	0.89	0.8	0.8	9.3	0.927	-0.5
$\delta^{18}O$	Species	*Ovis vel Capra*	*Unio tigridis*	6	4	-2.43	-0.26	-6.68	-2.02	-3.2	2.1	4.3	0.347	1.6
$\delta^{18}O$	Species	*Sus domesticus*	*Unio tigridis*	6	4	-2.08	0.89	-6.68	-2.02	-4.0	2.5	5.2	0.219	1.7
$\delta^{18}O$	Age	Adult	Non-Adult	16	15	-4.42	-2.83	-4.05	-2.59	0.3	0.5	28.9	0.982	-0.2

$\delta^{18}O$	Building	Building A1	Building A2	5	11	-4.52	-3.46	-4.42	-3.66	-0.1	0.1	8.0	0.999	0.1
$\delta^{18}O$	Building	Building A1	Cemetery Area 1	5	7	-4.52	-3.46	-5.23	-2.84	-0.1	0.1	8.2	1.000	0.1
$\delta^{18}O$	Building	Building A2	Cemetery Area 1	11	7	-4.42	-3.66	-5.23	-2.84	0.0	0.0	7.2	1.000	0.0
$\delta^{18}O$	Alimentation	Mother	Probable Exclusive Breastfeeding	7	4	-3.44	-0.20	-3.29	-0.48	0.1	0.1	8.5	1.000	-0.1
$\delta^{18}O$	Alimentation	Mother	Post lactation	7	11	-3.44	-0.20	-1.75	0.27	1.3	1.2	10.0	0.912	-0.6
$\delta^{18}O$	Alimentation	Mother	Post weaning	7	12	-3.44	-0.20	-4.58	-2.61	-1.7	1.6	10.1	0.758	0.8
$\delta^{18}O$	Alimentation	Mother	Weaning	7	3	-3.44	-0.20	-4.18	-1.53	-1.2	1.0	6.6	0.966	0.5
$\delta^{18}O$	Alimentation	Mother	Probable Post weaning	7	4	-3.44	-0.20	-5.52	-4.62	-3.1	3.1	7.2	0.155	1.5
$\delta^{18}O$	Alimentation	Probable Exclusive Breastfeeding	Post lactation	4	11	-3.29	-0.48	-1.75	0.27	1.2	1.2	5.7	0.910	-0.7
$\delta^{18}O$	Alimentation	Probable Exclusive Breastfeeding	Post weaning	4	12	-3.29	-0.48	-4.58	-2.61	-1.8	1.8	5.7	0.637	1.0
$\delta^{18}O$	Alimentation	Probable Exclusive Breastfeeding	Weaning	4	3	-3.29	-0.48	-4.18	-1.53	-1.3	1.1	4.8	0.926	0.8
$\delta^{18}O$	Alimentation	Probable Exclusive Breastfeeding	Probable Post weaning	4	4	-3.29	-0.48	-5.52	-4.62	-3.2	3.6	3.8	0.169	2.5
$\delta^{18}O$	Alimentation	Post lactation	Post weaning	11	12	-1.75	0.27	-4.58	-2.61	-3.0	3.9	21.0	0.014	1.6
$\delta^{18}O$	Alimentation	Post lactation	Weaning	11	3	-1.75	0.27	-4.18	-1.53	-2.5	2.5	3.8	0.386	1.5
$\delta^{18}O$	Alimentation	Post lactation	Probable Post weaning	11	4	-1.75	0.27	-5.52	-4.62	-4.4	7.1	13.0	<0.001	2.8
$\delta^{18}O$	Alimentation	Post weaning	Weaning	12	3	-4.58	-2.61	-4.18	-1.53	0.5	0.5	3.8	0.999	-0.3

$\delta^{18}O$	Alimentation	Post weaning	Probable Post weaning	12	4	-4.58	-2.61	-5.52	-4.62	-1.4	2.2	13.9	0.391	0.8
$\delta^{18}O$	Alimentation	Weaning	Probable Post weaning	3	4	-4.18	-1.53	-5.52	-4.62	-1.9	2.1	2.5	0.572	1.8
$\delta^{18}O$	Sex	F	M	6	8	-4.86	-1.86	-4.44	-2.16	0.3	0.3	9.4	0.990	-0.2
$\delta^{18}O$	Sex	F	ND	6	17	-4.86	-1.86	-4.19	-2.73	0.2	0.2	6.9	0.997	-0.1
$\delta^{18}O$	Sex	M	ND	8	17	-4.44	-2.16	-4.19	-2.73	-0.1	0.2	12.4	0.998	0.1
$\delta^{18}O$	Grave Goods	pottery vessels	pottery vessels, Reed basket	9	3	-4.31	-2.98	-4.53	-1.63	0.2	0.2	2.6	0.997	-0.2
$\delta^{18}O$	Grave Goods	pottery vessels	unknown	9	9	-4.31	-2.98	-4.34	-2.41	0.2	0.3	13.5	0.991	-0.1
$\delta^{18}O$	Grave Goods	pottery vessels, Reed basket	unknown	3	9	-4.53	-1.63	-4.34	-2.41	0.0	0.0	3.5	1.000	0.0
$\delta^{18}O$	Feeding Behaviour	Freshwater	Grazer	6	5	-5.07	0.13	-1.23	1.77	2.7	1.6	7.9	0.635	-0.9
$\delta^{18}O$	Feeding Behaviour	Freshwater	Human	6	29	-5.07	0.13	-4.14	-2.86	-1.0	0.7	5.5	0.976	0.5
$\delta^{18}O$	Feeding Behaviour	Freshwater	Omnivore	6	6	-5.07	0.13	-2.08	0.89	2.1	1.3	7.8	0.794	-0.7
$\delta^{18}O$	Feeding Behaviour	Freshwater	Ovicaprines	6	6	-5.07	0.13	-2.43	-0.26	1.3	0.8	6.7	0.952	-0.5
$\delta^{18}O$	Feeding Behaviour	Grazer	Human	5	29	-1.23	1.77	-4.14	-2.86	-3.7	4.0	5.1	0.061	2.1
$\delta^{18}O$	Feeding Behaviour	Grazer	Omnivore	5	6	-1.23	1.77	-2.08	0.89	-0.6	0.5	8.7	0.996	0.3
$\delta^{18}O$	Feeding Behaviour	Grazer	Ovicaprines	5	6	-1.23	1.77	-2.43	-0.26	-1.4	1.3	7.5	0.787	0.8
$\delta^{18}O$	Feeding Behaviour	Human	Omnivore	29	6	-4.14	-2.86	-2.08	0.89	3.1	3.6	6.6	0.066	-1.8
$\delta^{18}O$	Feeding Behaviour	Human	Ovicaprines	29	6	-4.14	-2.86	-2.43	-0.26	2.3	3.4	7.9	0.070	-1.4
$\delta^{18}O$	Feeding Behaviour	Omnivore	Ovicaprines	6	6	-2.08	0.89	-2.43	-0.26	-0.8	0.8	9.3	0.962	0.5
$\delta^{18}O$	Drinking Strategy	Freshwater	Non-Obligate Drinker	6	7	-5.07	0.13	-2.07	0.72	1.9	1.1	7.7	0.783	-0.7

$\delta^{18}O$	Drinking Strategy	Freshwater	Obligate Drinker	6	10	-5.07	0.13	-1.44	0.57	2.1	1.3	6.4	0.683	-0.8
$\delta^{18}O$	Drinking Strategy	Human	Non-Obligate Drinker	29	7	-4.14	-2.86	-2.07	0.72	2.9	3.5	8.1	0.045	-1.7
$\delta^{18}O$	Drinking Strategy	Human	Obligate Drinker	29	10	-4.14	-2.86	-1.44	0.57	3.1	5.0	15.6	0.001	-1.8
$\delta^{18}O$	Drinking Strategy	Non-Obligate Drinker	Obligate Drinker	7	10	-2.07	0.72	-1.44	0.57	0.2	0.2	11.5	1.000	-0.1

Table S8. Zinc, carbon, and oxygen isotope statistical tests and 95% confidence intervals for all groups from Abu Tbeirah, including all individuals. (ND = Non Determined)

Isotope	Category	Group 1	Group 2	n 1	n 2	G1 CIs Lower	G1 CIs Upper	G2 CIs Lower	G2 CIs Upper	Estimate	Statistic	df	P.adj	Effect Size
$\delta^{66}Zn$	Species	*Homo sapiens*	*Ovis vel Capra*	29	4	0.67	0.72	1.26	1.27	0.6	37.0	31.0	<0.001	-7.6
$\delta^{66}Zn$	Species	*Homo sapiens*	*Sus domesticus*	29	4	0.67	0.72	0.79	0.82	0.1	6.2	18.7	<0.001	-1.5
$\delta^{66}Zn$	Species	*Ovis vel Capra*	*Sus domesticus*	4	4	1.26	1.27	0.79	0.82	-0.5	39.9	4.2	<0.001	28.2
$\delta^{66}Zn$	Age	Adult	Non-Adult	15	15	0.65	0.71	0.68	0.79	0.1	1.7	22.1	0.228	-0.6
$\delta^{66}Zn$	Building	Animal	Building A1	18	3	0.92	1.15	0.68	0.73	-0.3	5.3	18.5	<0.001	1.4
$\delta^{66}Zn$	Building	Animal	Building A2	18	10	0.92	1.15	0.62	0.67	-0.4	6.3	18.8	<0.001	1.9
$\delta^{66}Zn$	Building	Animal	Cemetery Area 1	18	7	0.92	1.15	0.7	0.74	-0.3	5.2	18.4	<0.001	1.4
$\delta^{66}Zn$	Building	Building A1	Building A2	3	10	0.68	0.73	0.62	0.67	-0.1	3.1	6.2	0.073	1.5
$\delta^{66}Zn$	Building	Building A1	Cemetery Area 1	3	7	0.68	0.73	0.7	0.74	0.0	0.6	5.1	0.921	-0.4
$\delta^{66}Zn$	Building	Building A2	Cemetery Area 1	10	7	0.62	0.67	0.7	0.74	0.1	4.0	14.9	0.006	-1.9
$\delta^{66}Zn$	Alimentation	Mother	Probable Exclusive Breastfeeding	7	5	0.69	0.85	0.62	0.9	0.0	0.0	6.6	1.000	0.0
$\delta^{66}Zn$	Alimentation	Mother	Post lactation	7	11	0.69	0.85	0.9	1.21	0.3	3.0	14.7	0.060	-1.2
$\delta^{66}Zn$	Alimentation	Mother	Post weaning	7	13	0.69	0.85	0.64	0.69	-0.1	2.1	7.2	0.306	1.2

δ66Zn	Alimentation	Mother	Probable Post weaning	7	3	0.69	0.85	0.7	0.75	0.0	0.9	7.1	0.876	0.4
δ66Zn	Alimentation	Probable Exclusive Breastfeeding	Post lactation	5	11	0.62	0.9	0.9	1.21	0.3	2.5	11.8	0.166	-1.1
δ66Zn	Alimentation	Probable Exclusive Breastfeeding	Post weaning	5	13	0.62	0.9	0.64	0.69	-0.1	1.3	4.3	0.726	1.0
δ66Zn	Alimentation	Probable Exclusive Breastfeeding	Probable Post weaning	5	3	0.62	0.9	0.7	0.75	0.0	0.6	4.3	0.975	0.3
δ66Zn	Alimentation	Post lactation	Post weaning	11	13	0.9	1.21	0.64	0.69	-0.4	4.6	10.6	0.006	2.0
δ66Zn	Alimentation	Post lactation	Probable Post weaning	11	3	0.9	1.21	0.7	0.75	-0.3	3.9	10.6	0.018	1.3
δ66Zn	Alimentation	Post weaning	Probable Post weaning	13	3	0.64	0.69	0.7	0.75	0.1	2.7	6.5	0.154	-1.2
δ66Zn	Sex	F	M	5	7	0.62	0.68	0.63	0.74	0.0	1.1	8.7	0.705	-0.6
δ66Zn	Sex	F	ND	5	17	0.62	0.68	0.68	0.75	0.1	2.5	16.6	0.102	-0.8
δ66Zn	Sex	M	ND	7	17	0.63	0.74	0.68	0.75	0.0	0.7	11.5	0.892	-0.3
δ66Zn	Grave Goods	No	pottery vessels	4	8	0.69	0.8	0.66	0.74	-0.1	1.5	6.2	0.600	0.9
δ66Zn	Grave Goods	No	pottery vessels, Reed basket	4	3	0.69	0.8	0.61	0.73	-0.1	1.7	4.7	0.488	1.3
δ66Zn	Grave Goods	No	unknown	4	8	0.69	0.8	0.61	0.67	-0.1	3.2	4.6	0.121	2.2
δ66Zn	Grave Goods	pottery vessels	pottery vessels, Reed basket	8	3	0.66	0.74	0.61	0.73	0.0	0.5	4.1	0.978	0.3
δ66Zn	Grave Goods	pottery vessels	unknown	8	8	0.66	0.74	0.61	0.67	-0.1	2.0	12.6	0.342	1.0
δ66Zn	Grave Goods	pottery vessels, Reed basket	unknown	3	8	0.61	0.73	0.61	0.67	0.0	0.9	3.0	0.884	0.7

δ66Zn	Feeding Behaviour	Grazer	Human	4	29	0.92	1.06	0.67	0.72	-0.3	6.6	3.8	0.011	3.7
δ66Zn	Feeding Behaviour	Grazer	Omnivore	4	5	0.92	1.06	0.76	0.82	-0.2	4.4	4.0	0.039	3.2
δ66Zn	Feeding Behaviour	Grazer	Ovicaprines	4	4	0.92	1.06	1.26	1.27	0.3	6.4	3.1	0.021	-4.6
δ66Zn	Feeding Behaviour	Human	Omnivore	29	5	0.67	0.72	0.76	0.82	0.1	4.3	11.2	0.006	-1.3
δ66Zn	Feeding Behaviour	Human	Ovicaprines	29	4	0.67	0.72	1.26	1.27	0.6	37.0	31.0	<0.001	-7.6
δ66Zn	Feeding Behaviour	Omnivore	Ovicaprines	5	4	0.76	0.82	1.26	1.27	0.5	26.7	4.6	<0.001	-16.0
δ66Zn	Drinking Strategy	Human	Non-Obligate Drinker	29	6	0.67	0.72	1.23	1.28	0.6	27.4	16.9	<0.001	-7.7
δ66Zn	Drinking Strategy	Human	Obligate Drinker	29	9	0.67	0.72	0.81	0.95	0.2	4.3	10.3	0.004	-2.0
δ66Zn	Drinking Strategy	Non-Obligate Drinker	Obligate Drinker	6	9	1.23	1.28	0.81	0.95	-0.4	9.1	10.0	<0.001	4.0
δ13C	Species	*Homo sapiens*	Ovis vel Capra	28	4	-11.73	-11.37	-6.47	-5.72	5.5	22.1	4.118	<0.001	-11.0
δ13C	Species	*Homo sapiens*	Sus domesticus	28	6	-11.73	-11.37	-10.66	-8.78	1.9	3.5	5.33	0.074	-2.7
δ13C	Species	*Homo sapiens*	Unio tigridis	28	4	-11.73	-11.37	-7.52	-4.00	5.7	5.9	3.058	0.036	-7.4
δ13C	Species	*Ovis vel Capra*	*Sus domesticus*	4	6	-6.47	-5.72	-10.66	-8.78	-3.6	6.2	6.664	0.003	3.4
δ13C	Species	*Ovis vel Capra*	*Unio tigridis*	4	4	-6.47	-5.72	-7.52	-4.00	0.3	0.3	3.334	0.998	-0.2
δ13C	Species	*Sus domesticus*	*Unio tigridis*	6	4	-10.66	-8.78	-7.52	-4.00	3.9	3.5	4.795	0.085	-2.5
δ13C	Age	Adult	Non-Adult	15	14	-11.77	-11.26	-11.78	-11.25	0.0	0.1	26.81	1.000	0.0
δ13C	Building	Building A1	Building A2	3	11	-11.67	-11.50	-11.55	-10.96	0.3	1.8	11.42	0.317	-0.6
δ13C	Building	Building A1	Cemetery Area 1	3	7	-11.67	-11.50	-11.95	-11.41	-0.1	0.8	7.264	0.873	0.3
δ13C	Building	Building A2	Cemetery Area 1	11	7	-11.55	-10.96	-11.95	-11.41	-0.4	2.0	15.89	0.243	0.9
δ13C	Alimentation	Mother	Probable Exclusive Breastfeeding	6	4	-11.73	-10.13	-12.09	-11.59	-0.7	1.4	6.011	0.823	0.8

$\delta^{13}C$	Alimentation	Mother	Post lactation	6	11	-11.73	-10.13	-8.48	-5.82	3.9	4.5	14.94	0.007	-1.9
$\delta^{13}C$	Alimentation	Mother	Post weaning	6	10	-11.73	-10.13	-11.71	-11.46	-0.5	1.0	5.204	0.964	0.6
$\delta^{13}C$	Alimentation	Mother	Weaning	6	3	-11.73	-10.13	-12.22	-10.72	-0.4	0.5	6.171	0.999	0.3
$\delta^{13}C$	Alimentation	Mother	Probable Post weaning	6	4	-11.73	-10.13	-12.43	-11.26	-0.7	1.2	7.967	0.907	0.7
$\delta^{13}C$	Alimentation	Probable Exclusive Breastfeeding	Post lactation	4	11	-12.09	-11.59	-8.48	-5.82	4.7	6.3	10.87	0.001	-2.2
$\delta^{13}C$	Alimentation	Probable Exclusive Breastfeeding	Post weaning	4	10	-12.09	-11.59	-11.71	-11.46	0.3	1.5	4.202	0.792	-1.0
$\delta^{13}C$	Alimentation	Probable Exclusive Breastfeeding	Weaning	4	3	-12.09	-11.59	-12.22	-10.72	0.4	0.8	2.525	0.980	-0.7
$\delta^{13}C$	Alimentation	Probable Exclusive Breastfeeding	Probable Post weaning	4	4	-12.09	-11.59	-12.43	-11.26	0.0	0.0	4.175	1.000	0.0
$\delta^{13}C$	Alimentation	Post lactation	Post weaning	11	10	-8.48	-5.82	-11.71	-11.46	-4.4	6.0	10.18	0.002	2.5
$\delta^{13}C$	Alimentation	Post lactation	Weaning	11	3	-8.48	-5.82	-12.22	-10.72	-4.3	5.1	11.25	0.005	1.9
$\delta^{13}C$	Alimentation	Post lactation	Probable Post weaning	11	4	-8.48	-5.82	-12.43	-11.26	-4.7	5.8	12.85	0.001	2.2
$\delta^{13}C$	Alimentation	Post weaning	Weaning	10	3	-11.71	-11.46	-12.22	-10.72	0.1	0.2	2.1	1.000	-0.3
$\delta^{13}C$	Alimentation	Post weaning	Probable Post weaning	10	4	-11.71	-11.46	-12.43	-11.26	-0.3	0.7	3.236	0.989	0.6
$\delta^{13}C$	Alimentation	Weaning	Probable Post weaning	3	4	-12.22	-10.72	-12.43	-11.26	-0.4	0.7	4.218	0.995	0.5
$\delta^{13}C$	Sex	F	M	6	7	-12.04	-11.11	-11.89	-11.30	0.0	0.1	8.455	0.999	0.1
$\delta^{13}C$	Sex	F	ND	6	15	-12.04	-11.11	-11.76	-11.32	0.0	0.0	6.925	1.000	0.0

$\delta^{13}C$	Sex	M	ND	7	15	-11.89	-11.30	-11.76	-11.32	0.1	0.3	12.1	0.993	-0.1
$\delta^{13}C$	Grave Goods	pottery vessels	pottery vessels, Reed basket	7	3	-11.93	-11.53	-11.97	-10.33	0.7	1.4	2.215	0.613	-1.4
$\delta^{13}C$	Grave Goods	pottery vessels	unknown	7	8	-11.93	-11.53	-11.50	-11.13	0.4	2.4	12.73	0.122	-1.3
$\delta^{13}C$	Grave Goods	pottery vessels, Reed basket	unknown	3	8	-11.97	-10.33	-11.50	-11.13	-0.3	0.6	2.188	0.921	0.6
$\delta^{13}C$	Feeding Behaviour	Freshwater	Grazer	6	5	-6.58	-3.47	-5.93	-3.24	0.3	0.3	8.994	1.000	-0.2
$\delta^{13}C$	Feeding Behaviour	Freshwater	Human	6	28	-6.58	-3.47	-11.73	-11.37	-6.7	7.7	5.121	0.004	7.0
$\delta^{13}C$	Feeding Behaviour	Freshwater	Omnivore	6	6	-6.58	-3.47	-10.66	-8.78	-4.8	4.8	8.25	0.011	2.7
$\delta^{13}C$	Feeding Behaviour	Freshwater	Ovicaprines	6	4	-6.58	-3.47	-6.47	-5.72	-1.2	1.4	5.673	0.732	0.7
$\delta^{13}C$	Feeding Behaviour	Grazer	Human	5	28	-5.93	-3.24	-11.73	-11.37	-7.0	8.8	4.115	0.005	8.9
$\delta^{13}C$	Feeding Behaviour	Grazer	Omnivore	5	6	-5.93	-3.24	-10.66	-8.78	-5.1	5.4	7.177	0.008	3.4
$\delta^{13}C$	Feeding Behaviour	Grazer	Ovicaprines	5	4	-5.93	-3.24	-6.47	-5.72	-1.6	1.9	4.645	0.505	1.1
$\delta^{13}C$	Feeding Behaviour	Human	Omnivore	28	6	-11.73	-11.37	-10.66	-8.78	1.9	3.5	5.33	0.093	-2.7
$\delta^{13}C$	Feeding Behaviour	Human	Ovicaprines	28	4	-11.73	-11.37	-6.47	-5.72	5.5	22.1	4.118	<0.001	-11.0
$\delta^{13}C$	Feeding Behaviour	Omnivore	Ovicaprines	6	4	-10.66	-8.78	-6.47	-5.72	3.6	6.2	6.664	0.004	-3.4
$\delta^{13}C$	Drinking Strategy	Freshwater	Non-Obligate Drinker	6	7	-6.58	-3.47	-6.70	-5.16	-1.1	1.1	7.438	0.782	0.7
$\delta^{13}C$	Drinking Strategy	Freshwater	Obligate Drinker	6	10	-6.58	-3.47	-9.37	-5.67	-2.7	2.1	13.65	0.292	1.0
$\delta^{13}C$	Drinking Strategy	Human	Non-Obligate Drinker	28	7	-11.73	-11.37	-6.70	-5.16	5.6	12.6	6.585	<0.001	-8.4
$\delta^{13}C$	Drinking Strategy	Human	Obligate Drinker	28	10	-11.73	-11.37	-9.37	-5.67	4.0	4.0	9.167	0.018	-2.5
$\delta^{13}C$	Drinking Strategy	Non-Obligate Drinker	Obligate Drinker	7	10	-6.70	-5.16	-9.37	-5.67	-1.6	1.5	12.13	0.594	0.6

δ^{18}O	Species	*Homo sapiens*	Ovis vel Capra	26	5	-4.36	-3.48	-1.29	-0.17	3.3	8.1	8.462	<0.001	-2.9
δ^{18}O	Species	*Homo sapiens*	Sus domesticus	26	5	-4.36	-3.48	-0.48	1.13	4.3	8.2	6.107	0.001	-3.7
δ^{18}O	Species	*Homo sapiens*	Unio tigridis	26	4	-4.36	-3.48	-6.68	-2.02	-0.4	0.3	3.18	0.998	0.3
δ^{18}O	Species	*Ovis* vel *Capra*	*Sus domesticus*	5	5	-1.29	-0.17	-0.48	1.13	1.0	1.7	7.206	0.498	-1.1
δ^{18}O	Species	*Ovis* vel *Capra*	*Unio tigridis*	5	4	-1.29	-0.17	-6.68	-2.02	-3.7	2.7	3.371	0.240	2.0
δ^{18}O	Species	*Sus domesticus*	*Unio tigridis*	5	4	-0.48	1.13	-6.68	-2.02	-4.7	3.3	3.738	0.135	2.4
δ^{18}O	Age	Adult	Non-Adult	13	13	-4.73	-4.07	-4.38	-3.26	0.6	1.8	19.88	0.383	-0.7
δ^{18}O	Building	Building A1	Building A2	5	11	-4.52	-3.46	-4.40	-3.63	-0.1	0.1	7.954	0.999	0.1
δ^{18}O	Building	Building A1	Cemetery Area 1	5	7	-4.52	-3.46	-5.25	-2.68	-0.1	0.1	8.179	1.000	0.1
δ^{18}O	Building	Building A2	Cemetery Area 1	11	7	-4.40	-3.63	-5.25	-2.68	0.0	0.0	7.165	1.000	0.0
δ^{18}O	Alimentatio n	Mother	Probable Exclusive Breastfeedin g	6	4	-3.83	-1.82	-3.29	-0.49	0.9	0.9	5.716	0.975	-0.6
δ^{18}O	Alimentatio n	Mother	Post lactation	6	9	-3.83	-1.82	-0.51	0.59	2.8	4.4	7.598	0.033	-2.5
δ^{18}O	Alimentatio n	Mother	Post weaning	6	10	-3.83	-1.82	-4.75	-4.19	-1.7	2.8	5.78	0.255	1.8
δ^{18}O	Alimentatio n	Mother	Weaning	6	3	-3.83	-1.82	-4.18	-1.53	-0.4	0.4	3.933	1.000	0.3
δ^{18}O	Alimentatio n	Mother	Probable Post weaning	6	4	-3.83	-1.82	-5.70	-4.62	-2.3	3.5	7.224	0.099	1.9
δ^{18}O	Alimentatio n	Probable Exclusive Breastfeedin g	Post lactation	4	9	-3.29	-0.49	-0.51	0.59	1.9	2.1	3.749	0.516	-1.6
δ^{18}O	Alimentatio n	Probable Exclusive Breastfeedin g	Post weaning	4	10	-3.29	-0.49	-4.75	-4.19	-2.6	3.0	3.219	0.282	2.7
δ^{18}O	Alimentatio n	Probable Exclusive	Weaning	4	3	-3.29	-0.49	-4.18	-1.53	-1.3	1.1	4.784	0.926	0.8

$\delta^{18}O$	Alimentation	Breastfeeding Probable Exclusive Breastfeeding	Probable Post weaning	4	4	-3.29	-0.49	-5.70	-4.62	-3.2	3.6	3.763	0.169	2.5
$\delta^{18}O$	Alimentation	Post lactation	Post weaning	9	10	-0.51	0.59	-4.75	-4.19	-4.5	13.4	12.49	<0.001	6.4
$\delta^{18}O$	Alimentation	Post lactation	Weaning	9	3	-0.51	0.59	-4.18	-1.53	-3.2	3.6	2.495	0.233	3.2
$\delta^{18}O$	Alimentation	Post lactation	Probable Post weaning	9	4	-0.51	0.59	-5.70	-4.62	-5.1	12.1	8.461	<0.001	6.3
$\delta^{18}O$	Alimentation	Post weaning	Weaning	10	3	-4.75	-4.19	-4.18	-1.53	1.2	1.4	2.144	0.800	-1.6
$\delta^{18}O$	Alimentation	Post weaning	Probable Post weaning	10	4	-4.75	-4.19	-5.70	-4.62	-0.6	1.8	4.783	0.659	1.1
$\delta^{18}O$	Alimentation	Weaning	Probable Post weaning	3	4	-4.18	-1.53	-5.70	-4.62	-1.9	2.1	2.514	0.572	1.8
$\delta^{18}O$	Sex	F	M	4	8	-4.54	-4.11	-4.41	-2.17	1.0	1.6	7.575	0.445	-0.7
$\delta^{18}O$	Sex	F	ND	4	15	-4.54	-4.11	-4.43	-3.42	0.4	1.5	17	0.475	-0.5
$\delta^{18}O$	Sex	M	ND	8	15	-4.41	-2.17	-4.43	-3.42	-0.5	0.8	9.89	0.851	0.4
$\delta^{18}O$	Grave Goods	pottery vessels	pottery vessels, Reed basket	9	3	-4.34	-3.00	-4.53	-1.63	0.2	0.2	2.583	0.997	-0.2
$\delta^{18}O$	Grave Goods	pottery vessels	unknown	9	8	-4.34	-3.00	-4.48	-3.60	-0.3	0.7	13.95	0.880	0.4
$\delta^{18}O$	Grave Goods	pottery vessels, Reed basket	unknown	3	8	-4.53	-1.63	-4.48	-3.60	-0.5	0.5	2.281	0.945	0.5
$\delta^{18}O$	Feeding Behaviour	Freshwater	Grazer	6	5	-5.15	0.10	-1.36	1.64	2.7	1.6	7.91	0.635	-0.9
$\delta^{18}O$	Feeding Behaviour	Freshwater	Human	6	26	-5.15	0.10	-4.36	-3.48	-1.5	1.0	5.254	0.907	0.8
$\delta^{18}O$	Feeding Behaviour	Freshwater	Omnivore	6	5	-5.15	0.10	-0.48	1.13	2.8	1.8	6.015	0.505	-1.0
$\delta^{18}O$	Feeding Behaviour	Freshwater	Ovicaprines	6	5	-5.15	0.10	-1.29	-0.17	1.9	1.2	5.518	0.804	-0.7

$\delta^{18}O$	Feeding Behaviour	Grazer	Human	5	26	-1.36	1.64	-4.36	-3.48	-4.1	4.6	4.59	0.044	3.1
$\delta^{18}O$	Feeding Behaviour	Grazer	Omnivore	5	5	-1.36	1.64	-0.48	1.13	0.2	0.2	6.182	1.000	-0.1
$\delta^{18}O$	Feeding Behaviour	Grazer	Ovicaprines	5	5	-1.36	1.64	-1.29	-0.17	-0.8	0.9	5.165	0.939	0.6
$\delta^{18}O$	Feeding Behaviour	Human	Omnivore	26	5	-4.36	-3.48	-0.48	1.13	4.3	8.2	6.107	0.001	-3.7
$\delta^{18}O$	Feeding Behaviour	Human	Ovicaprines	26	5	-4.36	-3.48	-1.29	-0.17	3.3	8.1	8.462	<0.001	-2.9
$\delta^{18}O$	Feeding Behaviour	Omnivore	Ovicaprines	5	5	-0.48	1.13	-1.29	-0.17	-1.0	1.7	7.206	0.580	1.1
$\delta^{18}O$	Drinking Strategy	Freshwater	Non-Obligate Drinker	6	5	-5.15	0.10	-1.29	-0.15	1.9	1.2	5.518	0.732	-0.7
$\delta^{18}O$	Drinking Strategy	Freshwater	Obligate Drinker	6	9	-5.15	0.10	-0.81	0.76	2.5	1.6	5.814	0.534	-1.0
$\delta^{18}O$	Drinking Strategy	Human	Non-Obligate Drinker	26	5	-4.36	-3.48	-1.29	-0.15	3.3	8.1	8.462	<0.001	-2.9
$\delta^{18}O$	Drinking Strategy	Human	Obligate Drinker	26	9	-4.36	-3.48	-0.81	0.76	3.9	8.3	13.36	<0.001	-3.3
$\delta^{18}O$	Drinking Strategy	Non-Obligate Drinker	Obligate Drinker	5	9	-1.29	-0.15	-0.81	0.76	0.6	1.1	11.81	0.781	-0.6

Table S9. Zinc, carbon, and oxygen isotope statistical tests and 95% confidence intervals for different groups from Abu Tbeirah, excluding statistical outliers (defined as values beyond 1.5× the interquartile range). (ND = Non Determined)

Group	Variable 1	Variable 2	R^2	Adj R^2	F statistic	p value
All	$\delta^{18}O$	Ba/Ca	0.22	0.21	13.28	<0.01
Human	$\delta^{18}O$	Ba/Ca	0.19	0.16	6.19	0.02
All	$\delta^{18}O$	Sr/Ca	0.26	0.24	16.20	<0.01
Human	$\delta^{18}O$	Sr/Ca	0.02	-0.02	0.50	0.48
All	$\delta^{13}C$	$\delta^{66}Zn$	0.43	0.42	31.24	<0.01
Human	$\delta^{13}C$	$\delta^{66}Zn$	0.09	0.05	2.43	0.13
All	$\delta^{13}C$	Ba/Ca	0.73	0.72	122.03	<0.01
Human	$\delta^{13}C$	Ba/Ca	0.01	-0.03	0.20	0.66
All	$\delta^{13}C$	Sr/Ca	0.70	0.69	106.49	<0.01
Human	$\delta^{13}C$	Sr/Ca	0.03	-0.01	0.83	0.37
All	$\delta^{66}Zn$	Ba/Ca	0.43	0.42	34.59	<0.01
Human	$\delta^{66}Zn$	Ba/Ca	0.00	-0.03	0.14	0.71
All	$\delta^{66}Zn$	Sr/Ca	0.49	0.48	43.86	<0.01
Human	$\delta^{66}Zn$	Sr/Ca	0.00	-0.03	0.08	0.78
All	Ba.Ca	Sr/Ca	0.70	0.70	118.58	<0.01
Human	Ba.Ca	Sr/Ca	0.02	-0.01	0.69	0.41

Table S10. R^2 linear correlation of Ba/Ca and Sr/Ca with the isotopic values calculated on human group and on the complete dataset.

5.1 CIs and Statistical tests

Statistical tests and 95% confidence intervals (CIs) are presented in Table S8 and S9. The inclusion of samples potentially affected by diagenetic alteration did not significantly affect the results. Instead, outcomes were more sensitive to the presence of statistical outliers, supporting the decision to retain potentially altered specimens in the dataset. This trend is also observed in the Sr/Ca ratio (Figure S5), but not in the Ba/Ca ratio (Figure S6), where several outliers correspond to specific subgroups (e.g., Cemetery A1, non-obligate drinkers, and Probable Exclusive Breastfeeding individuals).

5.1.1 Values by Life History Stage

Zinc isotope values (Figure S4)

- Mother and Probable Exclusive Breastfeeding groups showed statistically similar means, although the latter displayed a wider distribution, even after outlier removal.
- Mother and post-weaning groups also had comparable means, as confirmed by overlapping CIs.

- Weaning/post-weaning and post-weaning groups had statistically similar means, but CIs revealed that outliers strongly affected the distribution, suggesting coexistence of both late weaning and early post-weaning signatures.

Carbon isotope values (Figure S7)

- Mother and Probable Exclusive Breastfeeding groups showed no statistically significant differences, although the Mother group displayed a wider distribution, which disappeared after outlier removal.

- Mother and post-weaning groups presented comparable means and narrow CIs once outliers were removed.

- Weaning/post-weaning and post-weaning groups' means were statistically similar, as supported by overlapping CIs.

Oxygen isotope values (Figure S8)

- No statistically significant differences were observed between Mother and Probable Exclusive Breastfeeding groups, based on overlapping CIs.

- Mother and post-weaning groups had overlapping CIs in the full dataset. Broadly suggesting the reflection of adult values in the Mother group.

- The same pattern was observed between Mother and weaning/post-weaning groups.

- Weaning/post-weaning and post-weaning groups showed statistically similar means, but the overlap in CIs did not support statistical equivalence.

5.1.2 Spatial Comparisons (Buildings and Cemetery Areas)

Zinc isotope values (Figure S4)

- Significant differences in means were not observed among Buildings A1, A2, and Cemetery Area 1.

- After removing outliers, a statistically significant difference in the means of Building A2 and Cemetery Area 1 and Building A2 and A1 was supported based on non-overlapping CIs; the latter is not supported by the statistical tests (Table S9).

Carbon isotope values (Figure S7)

- No significant differences in means were found among spatial groups. Building A1 showed a wider distribution, which disappeared after outlier removal.

Oxygen isotope values (Figure S8)

- No significant differences were observed among spatial groups, as confirmed by overlapping CIs.

5.1.3 Faunal Data

Zinc isotope values (Figure S4)

- Excluding outliers resulted in narrower distributions across faunal groups.

- Statistically significant differences were observed between:

    - Grazers, ovicaprines, and omnivores

- Humans and omnivores
- Humans, pigs, and ovicaprines

Statistically different means are evident between Grazers and ovicaprines. It should be considered, however, the wide distribution of Grazers is driven by outliers.

Carbon isotope values (Figure S7)

- Statistically significant differences were observed between most groups, except between grazers and ovicaprines (overlapping CIs).
- When removing outliers, humans and omnivores have significantly similar means; however, this is not supported by CIs.
- Slight depletion in $\delta^{13}C$ values was observed in some contaminated pig and ovicaprine samples.

Oxygen isotope values (Figure S8)

- Humans' means differed significantly in $\delta^{18}O$ from omnivores, grazers, and ovicaprines (non-overlapping CIs). However, this is not completely supported by Table S8. Freshwater shells have statistically significant similar means compare to the other groups.
- No significant differences were observed among the faunal groups themselves.
- CIs suggest that only herbivore values may have been slightly influenced by contamination.

5.1.4 Water Consumption Groups

Zinc isotope values (Figure S4)

- Statistically significant differences were observed between the means of obligate and non-obligate drinkers, and between both groups and humans.

Carbon isotope values (Figure S7)

- No significant differences between obligate and non-obligate drinkers.
- Significant differences observed between humans and faunal groups in accordance with water consumption.

Oxygen isotope values (Figure S8)

- Humans showed statistically significant differences in $\delta^{18}O$ values from both obligate and non-obligate drinkers.
- No significant difference was observed between the two faunal groups themselves, as indicated by overlapping CIs.

5.1.5 Biological sex

Zinc isotope values (Figure S4)

- No statistically significant differences in the means were found when comparing human individuals in relation to their biological sex. Females have a wider distribution.

Carbon isotope values (Figure S7)

- Comparisons by biological sex revealed no statistically significant variation in the mean values.

Oxygen isotope values (Figure S8)

- No statistically significant differences in mean values were observed between male, female, and not determined (ND) individuals. Females, however, have a wider distribution.

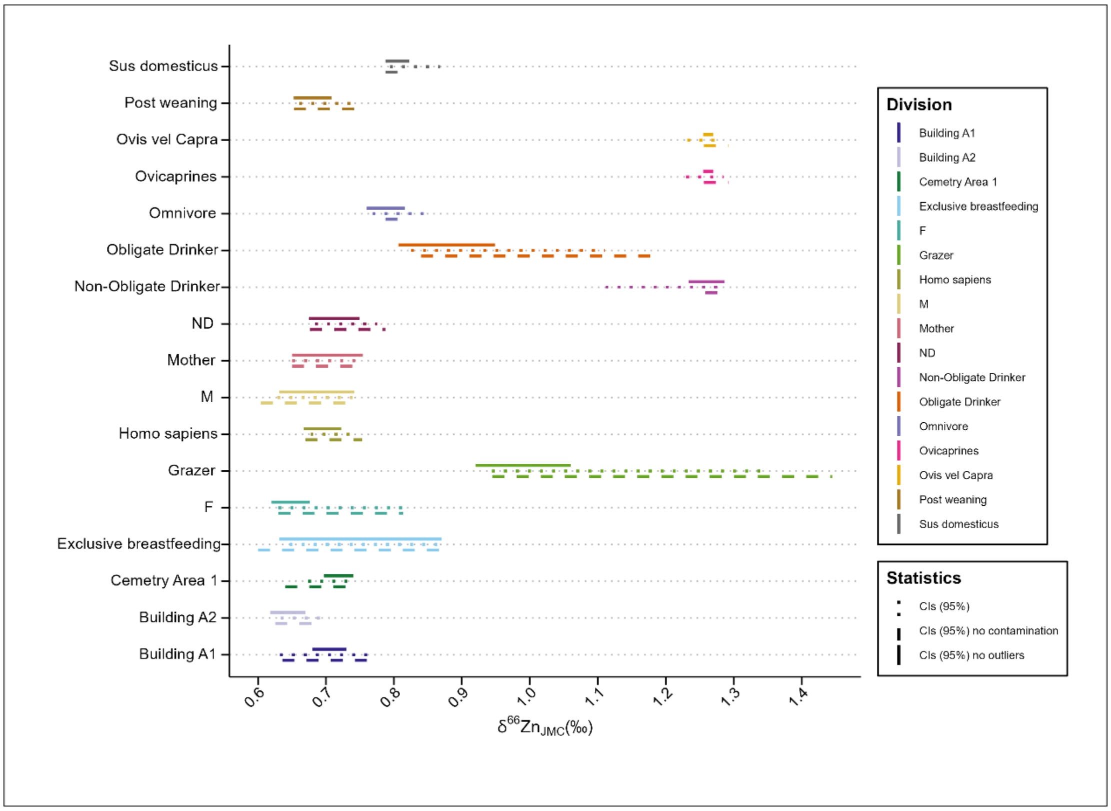

Figure S4. 95% confidence intervals (CIs) for δ^{66}Zn values grouped by tooth type, species, archaeological context, dietary category, and water-dependence (obligate vs non-obligate drinkers). Solid lines represent CIs excluding statistical outliers (1.5× IQR); dashed lines exclude potentially contaminated samples; dotted lines include all samples.

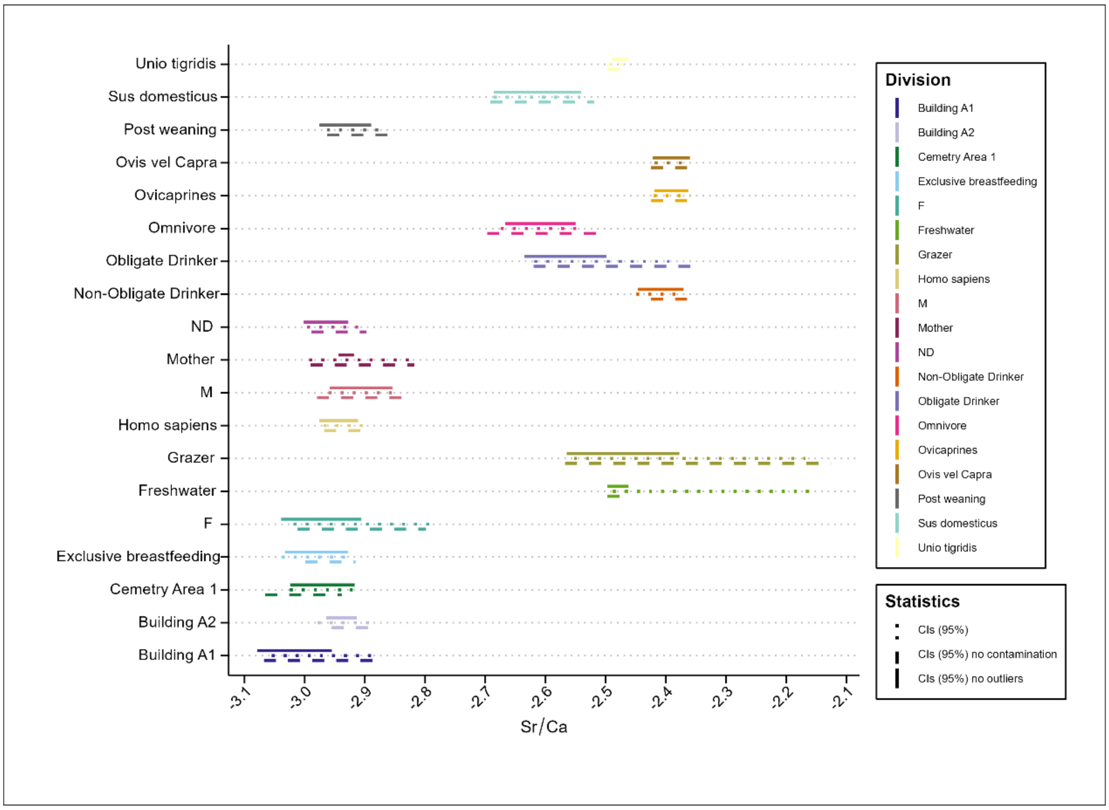

Figure S5. 95% confidence intervals (CIs) for Sr/Ca values grouped by tooth type, species, archaeological context, dietary category, and water-dependence (obligate vs non-obligate drinkers). Solid lines represent CIs excluding statistical outliers (1.5× IQR); dashed lines exclude potentially contaminated samples; dotted lines include all samples.

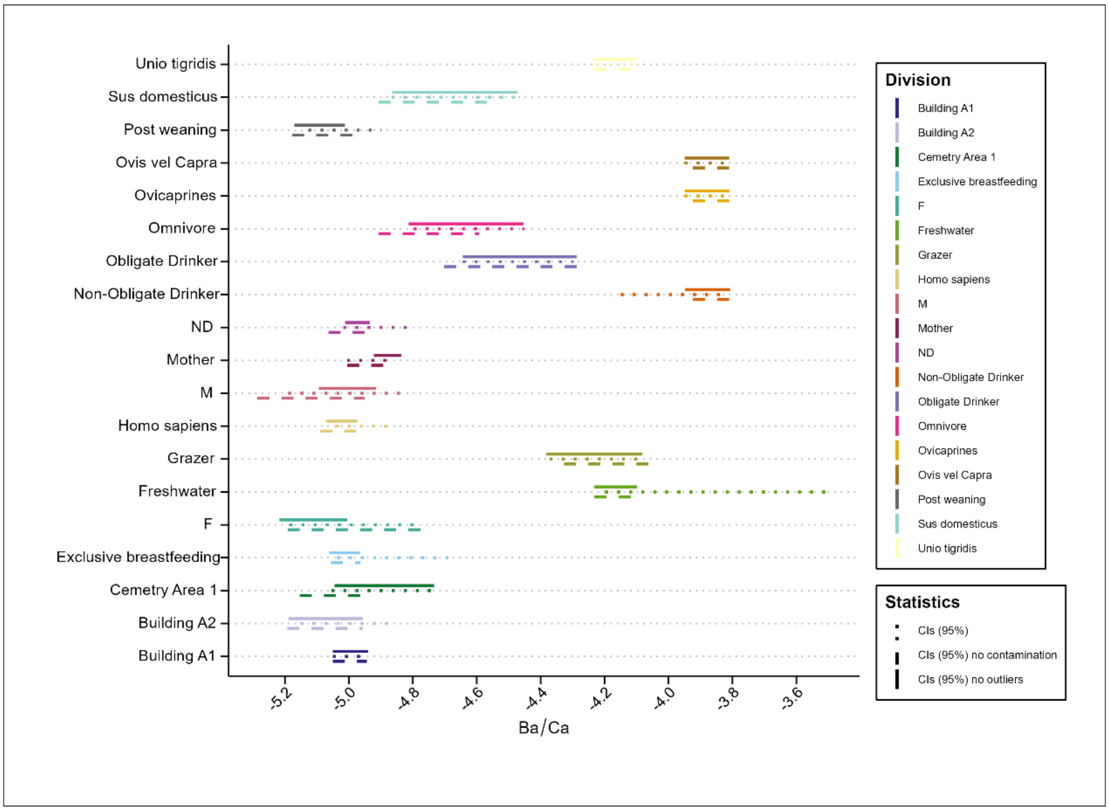

Figure S6. 95% confidence intervals (CIs) for Ba/Ca values grouped by tooth type, species, archaeological context, dietary category, and water-dependence (obligate vs non-obligate drinkers).

Solid lines represent CIs excluding statistical outliers (1.5× IQR); dashed lines exclude potentially contaminated samples; dotted lines include all samples.

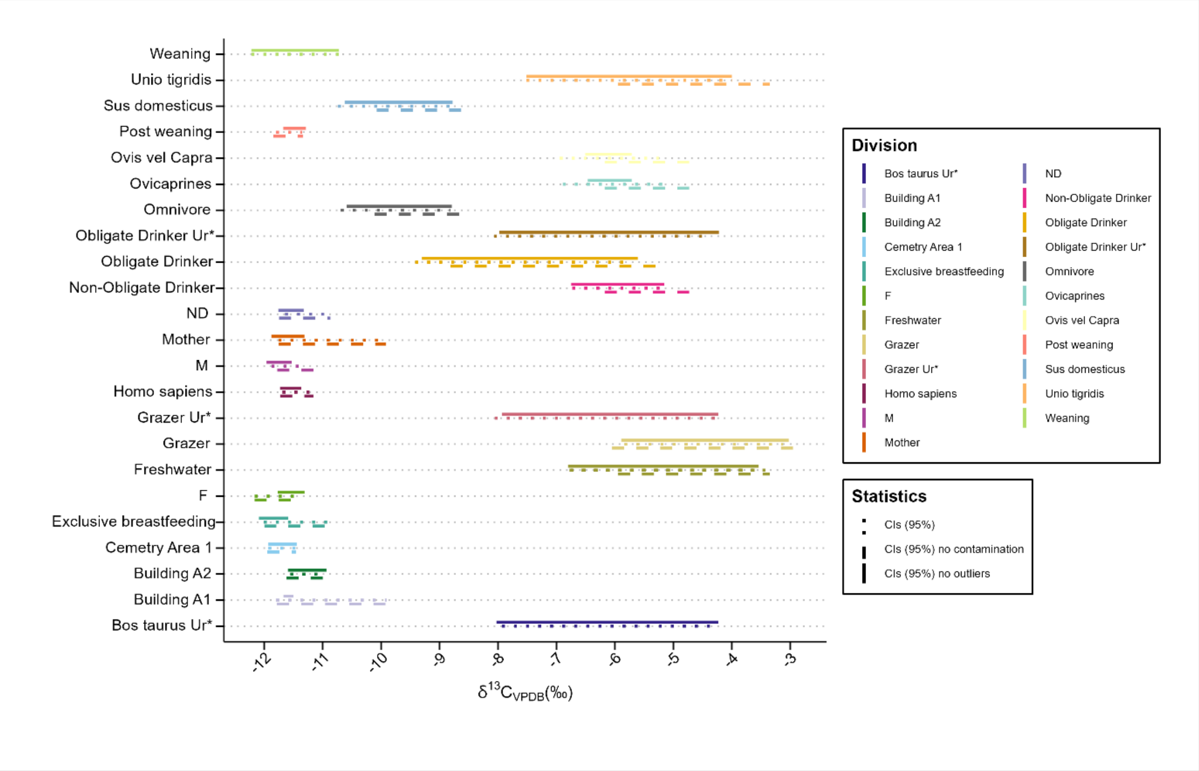

Figure S7. 95% confidence intervals (CIs) for δ^{13}C values grouped by tooth type, species, archaeological context, dietary category, and water-dependence (obligate vs non-obligate drinkers). Solid lines represent CIs excluding statistical outliers (1.5× IQR); dashed lines exclude potentially contaminated samples; dotted lines include all samples.

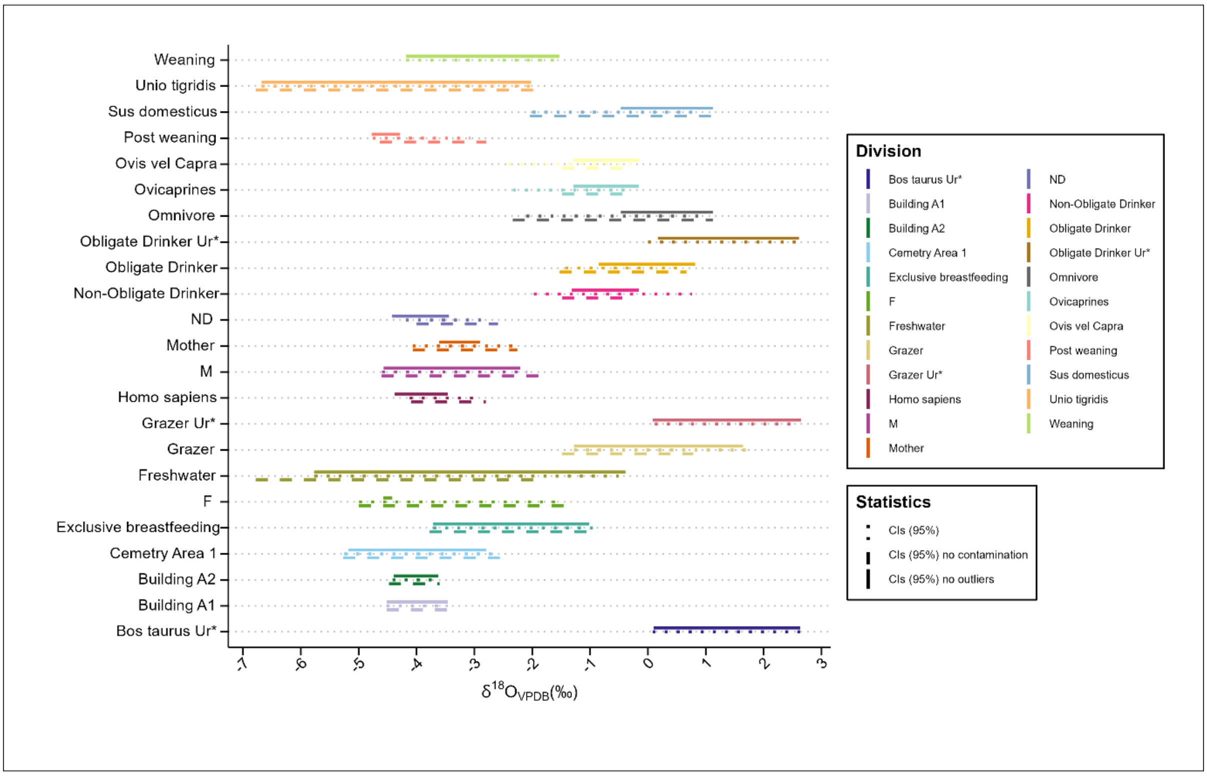

Figure S8. 5% confidence intervals (CIs) for δ^{18}O values grouped by tooth type, species, archaeological context, dietary category, and water-dependence (obligate vs non-obligate drinkers). Solid lines represent CIs excluding statistical outliers (1.5× IQR); dashed lines exclude potentially contaminated samples; dotted lines include all samples.

Supplementary figures

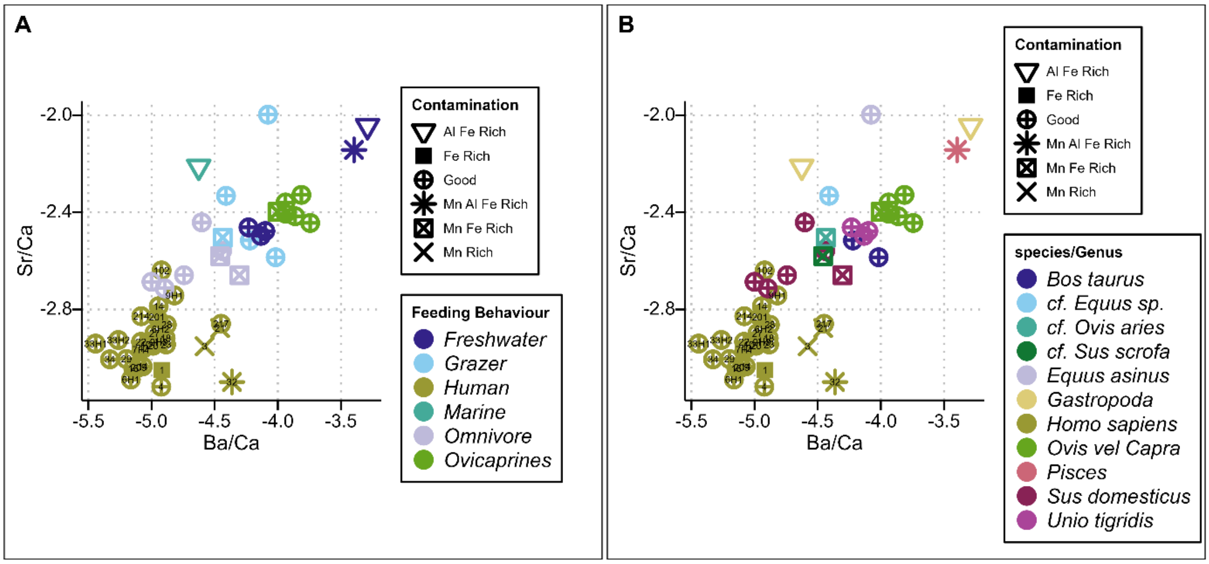

Figure S9. Scatterplot of Sr/Ca vs Ba/Ca ratios. (A) Points colored by dietary strategy; (B) points colored by species. In both panels, shape indicates contamination status based on trace element thresholds (Fe, Mn, Al). These plots assess trophic-level patterns and possible diagenetic overprints.

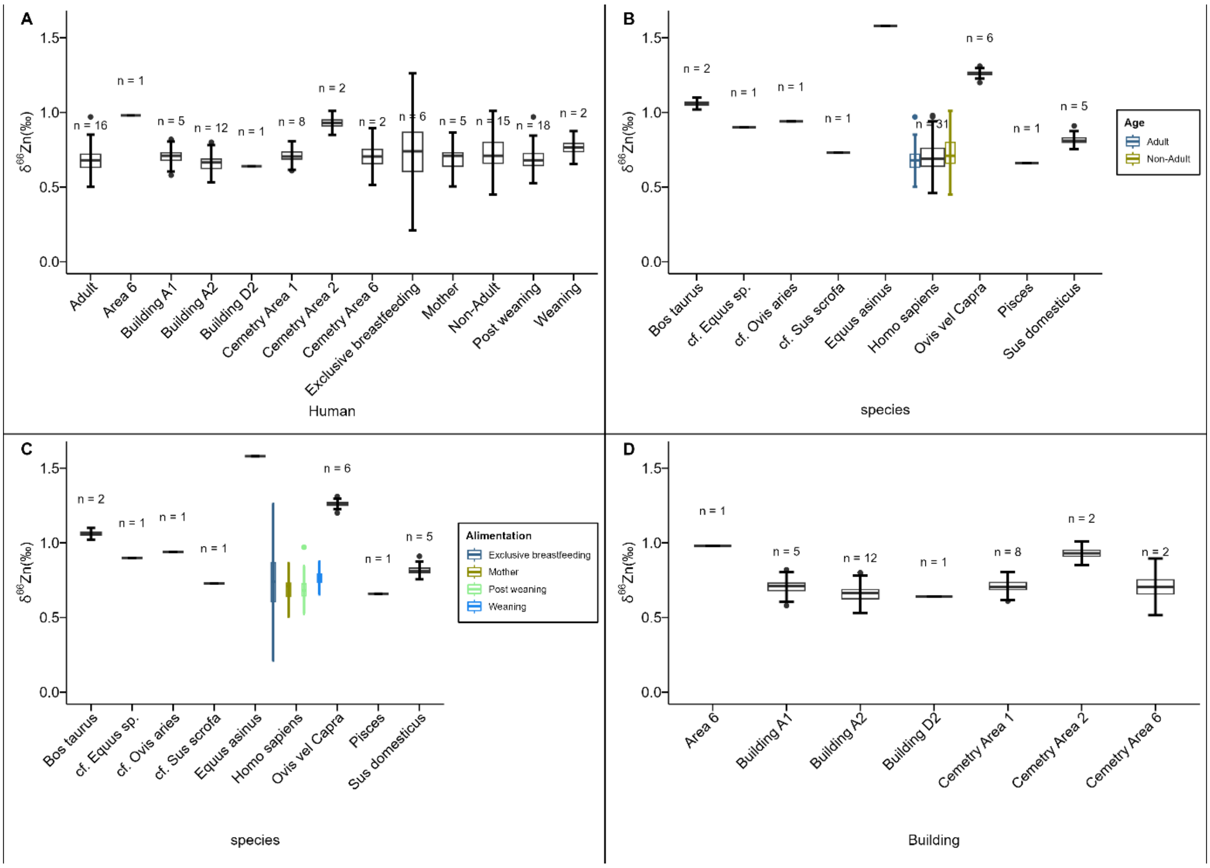

Figure S10. Boxplot of $\delta^{66}Zn$ values of individuals from Abu Tbeirah divided according to: A) Humans divided in the Building in which they were found and nutrition investigated through position of sampling; B) species and age of the humans; C) species and diet of humans; D) Building in which they were found. The fish values are likely from contamination.

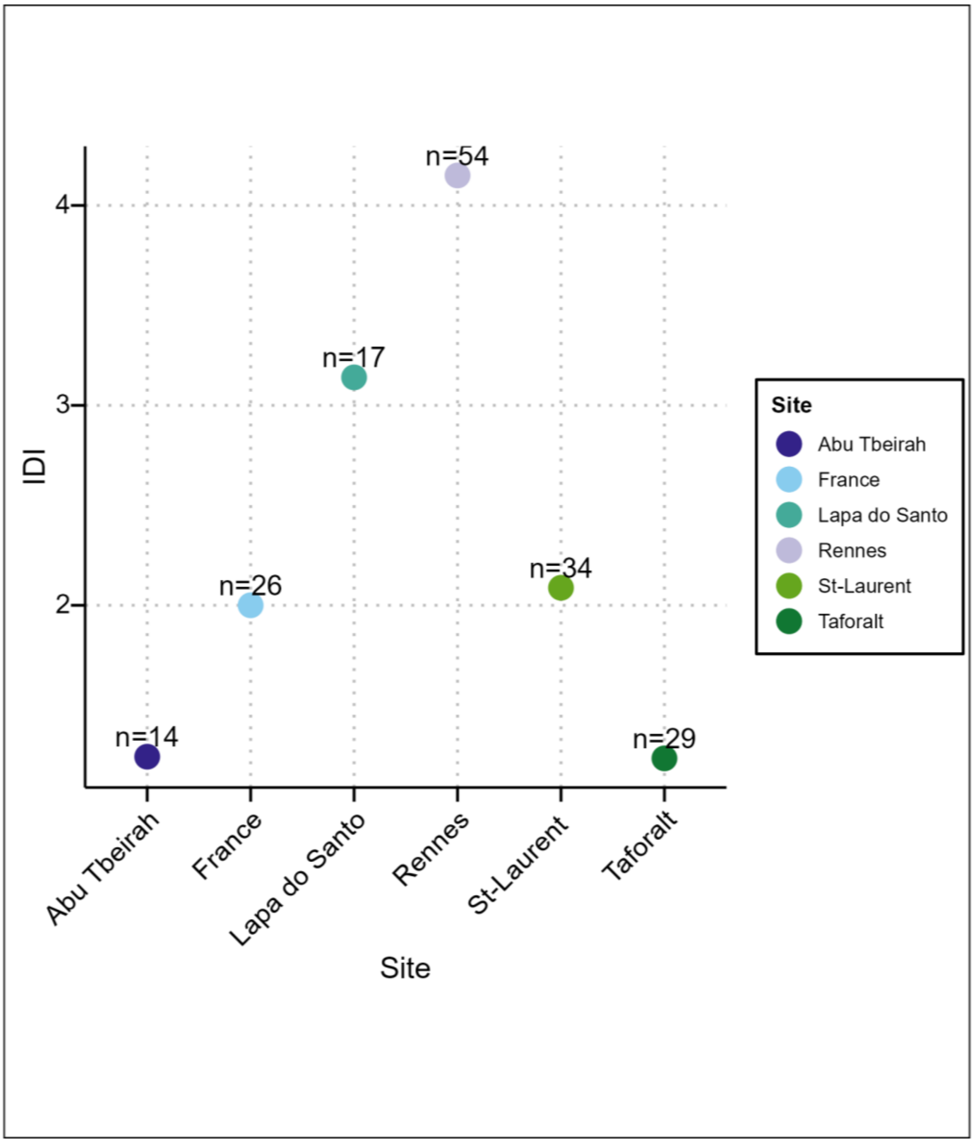

Figure S11. Interdecile index of available post-weaning zinc isotope values. Taforalt (36), Rennes (43), Lapa do Santo (42), Saint Laurent and France (75).

Supplementary Tables

Site	n	SD	Mean	Median	Minimum	Maximum	IQR	CI Lower	CI Upper	article
19th-century France	24	0.15	0.70	0.72	0.26	0.91	0.18	0.64	0.76	75
20th-century France	26	0.18	0.13	0.14	-0.18	0.69	0.12	0.07	0.21	75
Abu Tbeirah	14	0.06	0.67	0.68	0.58	0.80	0.07	0.64	0.70	current
Lapa do Santo	17	0.10	0.20	0.19	0.00	0.41	0.13	0.16	0.25	42
Rennes	54	0.21	0.39	0.36	0.00	1.04	0.24	0.33	0.45	43
St-Laurent	34	0.19	0.64	0.73	0.13	0.83	0.17	0.58	0.70	75
Taforalt	29	0.07	0.78	0.79	0.66	0.91	0.09	0.76	0.80	36

Table S11. Descriptive statistics of available post-weaning zinc isotope values.

SI References

1. A. Sołtysiak, H. Schutkowski, Continuity and change in subsistence at Tell Barri, NE Syria. J. Archaeol. Sci. Rep. 2, 176–185 (2015).
2. L. Romano, F. D'Agostino, Abu Tbeirah Excavations I. Area 1. Last Phase and Building A – Phase 1 (Sapienza Università Editrice, 2019).
3. E. R. Ellison, A Study of Diet in Mesopotamia (c. 3000-600 B.C.) and Associated Agricultural Techniques and Methods of Food Preparation (University of London, 1978).
4. E. J. Laugier, J. Casana, C. Glatz, S. M. Sameen, D. Cabanes, Reconstructing agro-pastoral practice in the Mesopotamian-Zagros borderlands: Insights from phytolith and FTIR analysis of a dung-rich deposit. J. Archaeol. Sci. Rep. 38, 103106 (2021).
5. J. Bottéro, The Oldest Cuisine in the World: Cooking in Mesopotamia (University of Chicago Press, 2011).
6. T. Wilkinson, M. Gibson, M. Widell, Models of Mesopotamian Landscapes: How Small-Scale Processes Contributed to the Growth of Early Civilizations (edited by T. J. Wilkinson, McGuire Gibson and Magnus Widell) (2013).
7. M. Widell, M. Gibson, T. Wilkinson, B. Studevent-Hickman, J. Tenney, "Household & Village in Early Mesopotamia" in (2013), pp. 112–129.
8. G. Scazzosi, Food and Drink Practices in Mesopotamian Households between the Early and Middle Bronze Ages. Università degli studi di Pavia (2018).
9. L. Jawad, Fishing Gear and Methods of the Lower Mesopotamian Plain with Reference to Fishing Management. Mar. Mesopotamica Online 1 (2006).
10. C. Stantis, H. Schutkowski, A. Sołtysiak, Reconstructing breastfeeding and weaning practices in the Bronze Age Near East using stable nitrogen isotopes. Am. J. Phys. Anthropol. 172, 58–69 (2020).
11. P. Wapnish, Animal Names and Animal Classifications in Mesopotamia: an Interdisciplinary Approach Based on Folk Taxonomy (UMI Dissertation Services, 1985).
12. F. D'Agostino, G. Spada, "Animal husbandry" in Society and the Individual in Ancient Mesopotamia, L. Culbertson, G. Rubio, Eds. (De Gruyter, 2024), pp. 147–178.
13. T. L. Greenfield, et al., Were there royal herds? Understanding herd management and mobility using isotopic characterizations of cattle tooth enamel from Early Dynastic Ur. PLOS ONE 17, e0265170 (2022).
14. F. D'Agostino, L. Romano, "Seven excavation campaigns at Abu Tbeirah" in Proceedings of the 11th International Congress on the Archaeology of the Ancient Near East: Vol. 2: Field Reports. Islamic Archaeology, Proceedings of the International Congress on the Archaeology of the Ancient Near East., 1st ed., A. Otto, M. Herles, K. Kaniuth, L. Korn, A. Heidenreich, Eds. (Harrassowitz Verlag, 2020), pp. 69–80.
15. F. D'Agostino, L. Romano, "Two new inscribed bricks from Abu Tbeirah (southern Iraq)" in The Third Millennium: Studies in Early Mesopotamia and Syria in Honor of Walter Sommerfeld and Manfred Krebernik, I. Arkhipov, L. Kogan, N. Koslova, Eds. (BRILL, 2020), pp. 259–269.
16. F. Alhaique, "Chapter 13: Faunal Remains" in Abu Tbeirah Excavations I. Area 1: Last Phase and Building A – Phase 1, (Sapienza Università Editrice, 2019), pp. 421–438.
17. F. Alhaique, L. Romano, F. D'Agostino, "Between sacred and profane: human–animal relationships at Abu Tbeirah (southern Iraq) in the third millennium BC" in Fierce Lions, Angry Mice and Fat-Tailed Sheep: Animal Encounters in the Ancient Near East, (McDonald Institute for Archaeological Research, 2021), pp. 63–78.
18. F. Alhaique, L. Romano, F. D'Agostino, "Ur-gir and the other dogs from Abu Tberah (Southern Iraq): Considerations on the role of dogs in Sumer during the 3rd Millennium BCE" in (Oxford, 2023).
19. V. Caleca, F. Alhaique, L. Romano, F. D'Agostino, "Sfruttamento delle risorse acquatiche ad Abu Tbeirah (Mesopotamia meridionale) nel III millennio a.C." in (Bologna, 2024).
20. S. Cereda, "Chapter 9: MICRO-DEBRIS ANALYSIS OF BUILDING A - PHASE 1 ROOM 23" in Abu Tbeirah Excavations I. Area 1: Last Phase and Building A – Phase 1, (Sapienza Università Editrice, 2019), pp. 421–438.
21. L. Forti, et al., The paleoenvironment and depositional context of the Sumerian site of Abu Tbeirah (Nasiriyah, southern Mesopotamia, Iraq). Quat. Res. 110, 165–183 (2022).
22. G. Iacobucci, et al., Combining Satellite Multispectral Imagery and Topographic Data for the Detection and Mapping of Fluvial Avulsion Processes in Lowland Areas. Remote Sens. 12, 2243 (2020).
23. T. J. Wilkinson, L. Rayne, J. Jotheri, Hydraulic landscapes in Mesopotamia: the role of human niche construction. Water Hist. 7, 397–418 (2015).

24. T. K. Al-Ameri, A. S. F. Al-Dolaymi, Human settlements adapted to environmental changes through the Paleolithic and Neolithic times in West Iraq. Arab. J. Geosci. 6, 2951–2960 (2013).
25. T. K. Al-Ameri, S. Y. Jassim, Environmental changes in the wetlands of Southern Iraq based on palynological studies. Arab. J. Geosci. 4, 443–461 (2011).
26. Y. T. Al-Rawi, B. S. Al-Tawash, T. K. Al-Ameri, POLLEN EVIDENCE OF LATE QUATERNARY VEGETATION AND INFERRED CLIMATIC CHANGES OF LAKE RAZZAZA, WESTERN IRAQI DESERT. IRAQI Bull. Geol. Min. 1, 1–13 (2005).
27. A. Kumar, Environmental changes in the wetlands of southern Iraq based on palynological studies: Comments. Arab. J. Geosci. 8, 4287–4289 (2015).
28. G. S. Morozova, A review of Holocene avulsions of the Tigris and Euphrates rivers and possible effects on the evolution of civilizations in lower Mesopotamia. Geoarchaeology 20, 401–423 (2005).
29. M. Engel, H. Brückner, "Chapter 3 Holocene Climate Variability of Mesopotamia and its Impact on the History of Civilisation" in (Brill, 2021).
30. S. H. Ambrose, Preparation and characterization of bone and tooth collagen for isotopic analysis. J. Archaeol. Sci. 17, 431–451 (1990).
31. M. A. Tafuri, "Chapter 12: The Human Remains" in Abu Tbeirah Excavations I. Area 1: Last Phase and Building A – Phase 1, (Sapienza Università Editrice, 2019), pp. 421–438.
32. J. Lee–Thorp, Two decades of progress towards understanding fossilization processes and isotopic signals in calcified tissue minerals. Archaeometry 44, 435–446 (2002).
33. B. Reynard, V. Balter, Trace elements and their isotopes in bones and teeth: Diet, environments, diagenesis, and dating of archeological and paleontological samples. Palaeogeogr. Palaeoclimatol. Palaeoecol. 416, 4–16 (2014).
34. K. Jaouen, What is our toolbox of analytical chemistry for exploring ancient hominin diets in the absence of organic preservation? Quat. Sci. Rev. 197, 307–318 (2018).
35. M. E. Fleet, Carbonated Hydroxyapatite: Materials, Synthesis, and Applications (Jenny Stanford Publishing, 2014).
36. Z. Moubtahij, et al., Isotopic evidence of high reliance on plant food among Later Stone Age hunter-gatherers at Taforalt, Morocco. Nat. Ecol. Evol. 8, 1035–1045 (2024).
37. N. Bourgon, et al., Dietary and homeostatic controls of Zn isotopes in rats: a controlled feeding experiment and modeling approach. Metallomics 16, mfae026 (2024).
38. K. Jaouen, et al., A Neandertal dietary conundrum: Insights provided by tooth enamel Zn isotopes from Gabasa, Spain. Proc. Natl. Acad. Sci. 119, e2109315119 (2022).
39. K. Jaouen, M. Beasley, M. Schoeninger, J.-J. Hublin, M. P. Richards, Zinc isotope ratios of bones and teeth as new dietary indicators: results from a modern food web (Koobi Fora, Kenya). Sci. Rep. 6, 26281 (2016).
40. N. Bourgon, et al., Zinc isotopes in Late Pleistocene fossil teeth from a Southeast Asian cave setting preserve paleodietary information. Proc. Natl. Acad. Sci. 117, 4675–4681 (2020).
41. S. Pederzani, et al., Stable isotopes show Homo sapiens dispersed into cold steppes ~45,000 years ago at Ilsenhöhle in Ranis, Germany. Nat. Ecol. Evol. 8, 578–588 (2024).
42. K. Jaouen, et al., Zinc isotope variations in archeological human teeth (Lapa do Santo, Brazil) reveal dietary transitions in childhood and no contamination from gloves. PLOS ONE 15, e0232379 (2020).
43. K. Jaouen, et al., Tracing intensive fish and meat consumption using Zn isotope ratios: evidence from a historical Breton population (Rennes, France). Sci. Rep. 8, 5077 (2018).
44. Moynier, D. Vance, T. Fujii, P. Savage, The Isotope Geochemistry of Zinc and Copper. Rev. Mineral. Geochem. 82, 543–600 (2017).
45. B. N. Smith, S. Epstein, Two Categories of 13C/12C Ratios for Higher Plants 1. Plant Physiol. 47, 380–384 (1971).
46. S. Jim, S. H. Ambrose, R. P. Evershed, Stable carbon isotopic evidence for differences in the dietary origin of bone cholesterol, collagen and apatite: implications for their use in palaeodietary reconstruction. Geochim. Cosmochim. Acta 68, 61–72 (2004).
47. S. H. Ambrose, L. Norr, "Experimental Evidence for the Relationship of the Carbon Isotope Ratios of Whole Diet and Dietary Protein to Those of Bone Collagen and Carbonate" in Prehistoric Human Bone: Archaeology at the Molecular Level, J. B. Lambert, G. Grupe, Eds. (Springer, 1993), pp. 1–37.
48. R. E. Stevens, S. Pederzani, K. Britton, S. K. Wexler, Bones and teeth isotopes as archives for palaeoclimatic, palaeoenvironmental and palaeoecological data. Quat. Sci. Rev. 357, 109320 (2025).
49. B. H. Passey, et al., Carbon isotope fractionation between diet, breath CO2, and bioapatite in

different mammals. J. Archaeol. Sci. 32, 1459–1470 (2005).
50. R. G. Harrison, M. A. Katzenberg, Paleodiet studies using stable carbon isotopes from bone apatite and collagen: examples from Southern Ontario and San Nicolas Island, California. J. Anthropol. Archaeol. 22, 227–244 (2003).
51. M. H. O'Leary, Carbon Isotopes in Photosynthesis: Fractionation techniques may reveal new aspects of carbon dynamics in plants. BioScience 38, 328–336 (1988).
52. E. Lightfoot, et al., Carbon and nitrogen isotopic variability in foxtail millet (Setaria italica) with watering regime. Rapid Commun. Mass Spectrom. 34, e8615 (2020).
53. E. Lightfoot, T. C. O'Connell, On the Use of Biomineral Oxygen Isotope Data to Identify Human Migrants in the Archaeological Record: Intra-Sample Variation, Statistical Methods and Geographical Considerations. PLOS ONE 11, e0153850 (2016).
54. M. Wallace, et al., Stable carbon isotope analysis as a direct means of inferring crop water status and water management practices. World Archaeol. 45, 388–409 (2013).
55. A. F. Diefendorf, K. E. Mueller, Scott. L. Wing, P. L. Koch, K. H. Freeman, Global patterns in leaf 13C discrimination and implications for studies of past and future climate. Proc. Natl. Acad. Sci. 107, 5738–5743 (2010).
56. Y. C. de Armas, A.-M. Mavridou, J. G. Domínguez, K. Hanson, J. Laffoon, Tracking breastfeeding and weaning practices in ancient populations by combining carbon, nitrogen and oxygen stable isotopes from multiple non-adult tissues. PLOS ONE 17, e0262435 (2022).
57. S. Pederzani, K. Britton, Oxygen isotopes in bioarchaeology: Principles and applications, challenges and opportunities. Earth-Sci. Rev. 188, 77–107 (2019).
58. J. Bryant, P. N. Froelich, A model of oxygen isotope fractionation in body water of large mammals. Geochim. Cosmochim. Acta 59, 4523–4537 (1995).
59. M. Pellegrini, J. Pouncett, M. Jay, M. P. Pearson, M. P. Richards, Tooth enamel oxygen "isoscapes" show a high degree of human mobility in prehistoric Britain. Sci. Rep. 6, 34986 (2016).
60. A. Serna, L. Prates, L. O. Valenzuela, D. C. Salazar-García, Back to the bases: Building a terrestrial water δ18O baseline for archaeological studies in North Patagonia (Argentina). Quat. Int. 548, 4–12 (2020).
61. R. Gonfiantini, M.-A. Roche, J.-C. Olivry, J.-C. Fontes, G. M. Zuppi, The altitude effect on the isotopic composition of tropical rains. Chem. Geol. 181, 147–167 (2001).
62. G. Faure, T. M. Mensing, Isotopes: Principles and Applications (Wiley, 2005).
63. A. Longinelli, Oxygen isotopes in mammal bone phosphate: A new tool for paleohydrological and paleoclimatological research? Geochim. Cosmochim. Acta 48, 385–390 (1984).
64. G. J. Bowen, et al., Stable hydrogen and oxygen isotope ratios of bottled waters of the world. Rapid Commun. Mass Spectrom. 19, 3442–3450 (2005).
65. M. J. Kohn, Predicting animal δ18O: Accounting for diet and physiological adaptation. Geochim. Cosmochim. Acta 60, 4811–4829 (1996).
66. N. E. Levin, T. E. Cerling, B. H. Passey, J. M. Harris, J. R. Ehleringer, A stable isotope aridity index for terrestrial environments. Proc. Natl. Acad. Sci. 103, 11201–11205 (2006).
67. S. Safont, A. Malgosa, M. e. Subirà, J. Gibert, Can trace elements in fossils provide information about palaeodiet? Int. J. Osteoarchaeol. 8, 23–37 (1998).
68. V. Balter, et al., Ecological and physiological variability of Sr/Ca and Ba/Ca in mammals of West European mid-Würmian food webs. Palaeogeogr. Palaeoclimatol. Palaeoecol. 186, 127–143 (2002).
69. J. H. Burton, L. E. Wright, Nonlinearity in the relationship between bone Sr/Ca and diet: Paleodietary implications. Am. J. Phys. Anthropol. 96, 273–282 (1995).
70. M. Sponheimer, D. de Ruiter, J. Lee-Thorp, A. Späth, Sr/Ca and early hominin diets revisited: new data from modern and fossil tooth enamel. J. Hum. Evol. 48, 147–156 (2005).
71. T. Tsutaya, M. Yoneda, Reconstruction of breastfeeding and weaning practices using stable isotope and trace element analyses: A review. Am. J. Phys. Anthropol. 156, 2–21 (2015).
72. N. Bourgon, et al., Trophic ecology of a Late Pleistocene early modern human from tropical Southeast Asia inferred from zinc isotopes. J. Hum. Evol. 161, 103075 (2021).
73. J. McCormack, et al., Zinc isotopes from archaeological bones provide reliable trophic level information for marine mammals. Commun. Biol. 4, 1–11 (2021).
74. D. Yeghicheyan, et al., A New Interlaboratory Characterisation of Silicon, Rare Earth Elements and Twenty-Two Other Trace Element Concentrations in the Natural River Water Certified Reference Material SLRS-6 (NRC-CNRC). Geostand. Geoanalytical Res. 43, 475–496 (2019).
75. K. Jaouen, E. Herrscher, V. Balter, Copper and zinc isotope ratios in human bone and enamel. Am. J. Phys. Anthropol. 162, 491–500 (2017).

76. R Core Team, R: A Language and Environment for Statistical Computing. R Foundation for Statistical Computing. (2025). Deposited 2025.
77. P. Vaiglova, N. A. Lazar, E. A. Stroud, E. Loftus, C. A. Makarewicz, Best practices for selecting samples, analyzing data, and publishing results in isotope archaeology. Quat. Int. 650, 86–100 (2023).
78. H. Wickham, et al., ggplot2: Create Elegant Data Visualisations Using the Grammar of Graphics. (2025). Deposited 9 April 2025.
79. H. Wickham, et al., dplyr: A Grammar of Data Manipulation. (2023). Deposited 17 November 2023.
80. F. E. Jr. Harrell, C. Dupont, Hmisc: Harrell Miscellaneous. (2025). Deposited 16 March 2025.
81. P. A. Games, J. F. Howell, Pairwise Multiple Comparison Procedures with Unequal N'S and/or Variances: A Monte Carlo Study. J. Educ. Stat. 1, 113–125 (1976).
82. B. L. Welch, On The Comparison Of Several Mean Values: An Alternative Approach. Biometrika. 38, 330–336 (1951).
83. M. Giaccari, et al., Diet in high mediaeval Florence through stable isotope analysis of carbon, nitrogen and sulphur. J. Archaeol. Sci. Rep. 59, 104783 (2024).
84. D. S. Moore, The practice of statistics for business and economics, 3rd ed., international ed (W.H. Freeman, 2011).
85. G. Cumming, Inference by eye: Reading the overlap of independent confidence intervals. Stat. Med. 28, 205–220 (2009).
86. J. J. Dziak, L. C. Dierker, B. Abar, The interpretation of statistical power after the data have been gathered. Curr. Psychol. 39, 870–877 (2020).
